# The genetic diversity and evolution of field pea (*Pisum*) studied by high throughput retrotransposon based insertion polymorphism (RBIP) marker analysis

**DOI:** 10.1186/1471-2148-10-44

**Published:** 2010-02-15

**Authors:** Runchun Jing, Alexander Vershinin, Jacek Grzebyta, Paul Shaw, Petr Smýkal, David Marshall, Michael J Ambrose, TH Noel Ellis, Andrew J Flavell

**Affiliations:** 1 Division of Plant Sciences, University of Dundee at SCRI, Invergowrie, DUNDEE 5DA, UK; 2 John Innes Centre, Colney, Norwich, NR4 7UH, UK; 3 Scottish Crop Research Institute, Invergowrie, Dundee, DD2 5DA, UK; 4 Agritec Plant Research Ltd, Plant Biotechnology Department, Zemědělská 2520/16, CZ-787 01 Šumperk, Czech Republic; 5 Current address: School of Biological Sciences, University of East Anglia, Norwich, NR4 7TJ, UK; 6 Current address: Institute of Cytology and Genetics, Novosibirsk, Russia; 7 Current address: Rothamstead Research, Harpenden, Herts, UK

## Abstract

**Background:**

The genetic diversity of crop species is the result of natural selection on the wild progenitor and human intervention by ancient and modern farmers and breeders. The genomes of modern cultivars, old cultivated landraces, ecotypes and wild relatives reflect the effects of these forces and provide insights into germplasm structural diversity, the geographical dimension to species diversity and the process of domestication of wild organisms. This issue is also of great practical importance for crop improvement because wild germplasm represents a rich potential source of useful under-exploited alleles or allele combinations. The aim of the present study was to analyse a major *Pisum *germplasm collection to gain a broad understanding of the diversity and evolution of *Pisum *and provide a new rational framework for designing germplasm core collections of the genus.

**Results:**

3020 *Pisum *germplasm samples from the John Innes *Pisum *germplasm collection were genotyped for 45 retrotransposon based insertion polymorphism (RBIP) markers by the Tagged Array Marker (TAM) method. The data set was stored in a purpose-built Germinate relational database and analysed by both principal coordinate analysis and a nested application of the Structure program which yielded substantially similar but complementary views of the diversity of the genus *Pisum*. Structure revealed three Groups (1-3) corresponding approximately to landrace, cultivar and wild *Pisum *respectively, which were resolved by nested Structure analysis into 14 Sub-Groups, many of which correlate with taxonomic sub-divisions of *Pisum*, domestication related phenotypic traits and/or restricted geographical locations. Genetic distances calculated between these Sub-Groups are broadly supported by principal coordinate analysis and these, together with the trait and geographical data, were used to infer a detailed model for the domestication of *Pisum*.

**Conclusions:**

These data provide a clear picture of the major distinct gene pools into which the genus *Pisum *is partitioned and their geographical distribution. The data strongly support the model of independent domestications for *P. sativum ssp abyssinicum *and *P. sativum*. The relationships between these two cultivated germplasms and the various sub-divisions of wild *Pisum *have been clarified and the most likely ancestral wild gene pools for domesticated *P. sativum *identified. Lastly, this study provides a framework for defining global *Pisum *germplasm which will be useful for designing core collections.

## Background

The genetic diversity of a species is the outcome of cumulative mutation, recombination and selection on individuals by the environment. Plant and animal species important in agriculture have been additionally subjected to thousands of years of selection and breeding for traits desirable for cultivation or consumption by people or domesticated animals. Initially, wild plants carrying promising traits for cultivation were selected, leading eventually to locally adapted landraces that had lost many allele combinations that were disadvantageous to the farmer such as dehiscent pods and thick testas and gained useful ones such as increased seed size [1]. Modern breeding has largely continued this process by crossing the 'best with the best' and the increases in yield and performance are still being maintained today. This strong selection has narrowed the genetic diversity of cultivated germplasm for at least some species [2].

Wild relatives of many crop plant species are still extant in their natural habitat. Understanding the detailed relationships between the genomes of modern cultivars, old cultivated landraces, ecotypes and wild relatives is intrinsically interesting as it reflects the effects of human intervention upon both cultivated and wild germplasm, the geographical dimension to species diversity and the process of domestication of wild organisms. This information is of great interest to those studying and preserving biodiversity and it provides new paradigms against which existing data sets and theories can be viewed. These issues are also of great practical importance because wild germplasm carries a wide diversity of alleles that represent a rich potential source of new alleles or allele combinations for crop improvement.

Assessment of the genetic diversity within crop species is typically performed by marker genotyping of samples selected from gene banks (germplasm collections). Until recently, marker technologies were unable to deal effectively with the very large sample numbers contained in such collections. Therefore, most genotypic analyses have been confined to subsets of germplasm collections that have been selected to represent the broader collections. The effectiveness of this approach depends upon the criteria used for selecting germplasm subsets. In the absence of molecular marker data, these have typically been based on morphology or ecogeography and have likely misrepresented the diversity contained within the collection. Fortunately, the advent of high throughput genotyping technologies has made the description of the genetic diversity of complete germplasm collections a feasible proposition. This raises the possibility of defining a large proportion of the complete diversity of the corresponding species.

Field pea (*Pisum sativum*) derives from the Middle East and was first cultivated roughly 10,000 years ago [3-5]. Cultivated *Pisum *is dominated by *P. sativum*, but *P. sativum *ssp *abyssinicum *(here referred to as *P. abyssinicum*) is an independently derived cultivated type [6,7]. *Pisum sativum *is widespread across the Middle East and has affinity with the wild *P. elatius *while *P. abyssinicum *is restricted to highland regions of Ethiopia and Southern Yemen and shows a greater affinity to *P. fulvum *[6]. *P. elatius *(taken to include other minor taxa) is distributed widely across the Mediterranean basin from Spain to the Middle East, while *P. fulvum *is found around its eastern edge (Syria, Lebanon, Israel, Palestine and Jordan) although herbarium specimens are known from Cyprus, Greece, Iraq, Turkey and the former Yugoslavia [8]. *Pisum *is perhaps best considered as a species complex with multiple sub-species which interbreed to different degrees [6]. In support of this, there is extensive sharing of retrotransposon markers between all *Pisum *species [6], suggesting that there has been significant outcrossing and introgression between them, despite the predominantly inbreeding nature of the genus (greater than 99%), which reduces gene flow. Most wide crosses within *Pisum *are fertile, except for crosses involving *P. fulvum*. *P. fulvum *also forms a distinct clade in all molecular diversity analyses [[6] and references therein] and is the only realistic candidate in the genus for a distinct species. *P. sativum *is nested within the diversity of *P. elatius *in most molecular analyses and may even be paraphyletic [6,7,9], suggesting that cultivated *P. sativum *derived mainly from the latter species. Other claimed wild species, such as *P. humile *and *P. jomardii *have little support from molecular studies. *P. abyssinicum *has been considered to be a subspecies of *P. sativum *[discussed in [6]] but it shares several characteristic phenotypic traits and a significant proportion of molecular marker alleles with *P. fulvum *and tends to sit between the latter and *P. elatius *in molecular diversity plots [6,10]. Microsatellite-based marker analysis suggests that Chinese *Pisum *germplasm is rather distinct from the global gene pool and includes several rare alleles [11,12]. Based upon these data a Chinese P. sativum core collection has been proposed [12].

The John Innes (JI) *Pisum *Collection [13] currently comprises 3512 accessions, representing 1200 *P. sativum *cultivars, 600 traditional landraces and 750 wild *Pisum *samples (mainly *P. sativum, P. fulvum *and *P elatius*), together with genetic stocks and reference lines from other collections. This constitutes perhaps the widest and most comprehensive set of *Pisum *germplasm worldwide. Initially formed to support a range of pea research and a breeding programme, the collection has continually sought to draw together the broadest range of cultivated forms from both cultivated genepools and wild and weedy forms. Significant numbers of accessions are the result of expeditions including those to the primary centre of diversity in the Middle East and important secondary centres in the highland regions of Ethiopia and Asiatic regions including the Hindu Kush [14]. This has made it an excellent candidate resource for studying the full diversity of the genus *Pisum *and this is the main reason it was selected for this study.

The retrotransposon markers used in the above studies are mainly based upon insertions of the *PDR1 *Ty1-*copia *group retrotransposon, visualized by the Sequence-Specific Amplification Polymorphisms (SSAP) technique [6]. SSAP is a useful method for scoring diversity in relatively small sample sets (up to roughly 50 samples corresponding to a single gel assay) but, like all gel-based marker approaches, has problems dealing with large sample numbers owing to problems relating the results (band sharing) from one gel to the next. Furthermore, SSAP markers are dominant, thus band absence can mean either PCR failure or absence of the insertion. Therefore, to genotype thousands of samples we developed the Retrotransposon-Based Insertion Polymorphism (RBIP) method [15]. RBIP uses simple PCR-based detection of the presence or absence of single transposon insertions by combining two primers flanking the insertion site with a single outward-priming transposon-specific primer [15-17,19,20]. RBIP has been used to deduce the insertional polymorphisms of retrotransposons in diverse rice varieties, providing evidence for independent domestication events for indica and japonica rice [20].

One concern when using retrotransposon markers is the possibility of non-random chromosomal distributions due to insertion site preference or selection against insertions at specific locations, which can for example lead to strong clustering in centromeric and pericentromeric heterochromatin. Such non-random distributions can differ between different elements and/or species and might distort inferred genetic relationships. Fortunately, Jing *et al*. [19] showed that, for *PDR1*, the element upon which the bulk of the RBIP markers analysed were based, the sequence specificity of insertion sites is very relaxed, and slightly more insertions than expected are associated with genic sequences. Furthermore, genetic mapping of *PDR1 *insertion sites [21] showed no marked clustering of polymorphic *PDR1 *insertion sites. Therefore, *PDR1*-based RBIP markers should be excellent for assessing genetic diversity in *Pisum*.

For high throughput analysis RBIP amplicons can be scored by the Tagged Microarray Marker (TAM) format, which is based upon fluorescent microarray marker scoring [16,17]. RBIP-TAM yields codominant marker scores which are particularly relevant for studying diversity at the genus level because they mutate at around the 10^-6 ^year level [19], which gives them high polymorphism relative to SNPs and homoplasy is not an issue because retrotransposon insertions are effectively irreversible. SNPs are also useful markers for diversity analyses but require investment in SNP discovery and the setup of a corresponding genotyping platform.

The aims of the present study were; i) To analyse the genetic diversity of virtually the complete JI *Pisum *Collection using RBIP TAM marker technology; ii) To gain a broad understanding of the relationships between wild and cultivated *Pisum *germplasm and the link between genetic diversity and geographical distribution of *Pisum*; iii) To make the marker and accession data freely available via a web-accessible relational database; iv) To create a diversity framework to aid the rational design of a global *Pisum *germplasm collection.

## Methods

### Plant Materials and DNA extraction

3020 accessions comprising 92% of the complete JI Pisum Collection at the time of sampling, together with 208 lines from a private breeding program and 35 lines in the process of inclusion included in the genebank were selected for analysis (Additional file 1) [13]. The only significant omissions from the Collection were three mapping populations comprising recombinant inbred lines prepared from JI accessions JI399 and JI15 or JI281 and JI 15 and JI 1194. All segregating RBIP markers were separately mapped in these populations (data not shown). Individual plants were grown on after tissue sampling for DNA preparation, selfed and seed was collected from the majority of lines to form an independent resource linked to the marker database. These lines and all germplasm maintained at the John Innes *Pisum *germplasm collection are freely available from the Genetic Resources Unit of the John Innes Centre [18]. DNAs were extracted by the Qiagen DNeasy 96 method.

### RBIP marker analysis

RBIP reactions, based on 78 polymorphic LTR retrotransposon insertions [19] and two small indel polymorphisms flanking the 261 × 1 and 2055nr53 insertions respectively, were carried out as described [17]. Briefly, PCRs were carried out in 384 well format with three primers, a common biotinylated primer which amplifies both occupied and unoccupied alleles, plus two allele-specific primers for the occupied and unoccupied alleles respectively, carrying tags allowing detection with fluorescently labelled tag detector probes (see below). All PCR reactions used Qiagen HotStar Taq DNA polymerase, Qiagen reaction buffer, 0.25 μM each oligonucleotide [17] and 200 μM dNTPs. High throughput PCRs were set up manually by multi-channel pipette (Finnpipette) and were on a 10 μl (occasional) or 5 μl (typical) scale in a Tetrad instrument (Bio-Rad), fitted with 4 × 384 well blocks, using 6 ng of template DNA. Cycling conditions for retrotransposon-based PCRs were typically 95°C 15 minutes (heat-activation of enzyme), followed by 40 cycles of 94°C, 55°C, 72°C, all for 1 minute, with a final elongation step of 72°C for 7 min. Exceptions to the above 55°C annealing temperature were as follows; 2055 × 36 (57°C), 2055nr16 (60°C), 2055nr23 (60°C), 1006nr27, (58°C), 1006nr32 (60°C), 45 × 31 (60°C), 45 × 33 (60°C), 45 × 8 (60°C), 2539 × 7 (57°C), 3150 × 11 (60°C), MKRBIP2 (54°C), MKRBIP3 (54°C), MKRBIP4 (54°C), MKRBIP7 (54°C).

### TAM microarray-based marker analysis

**Figure 1:**
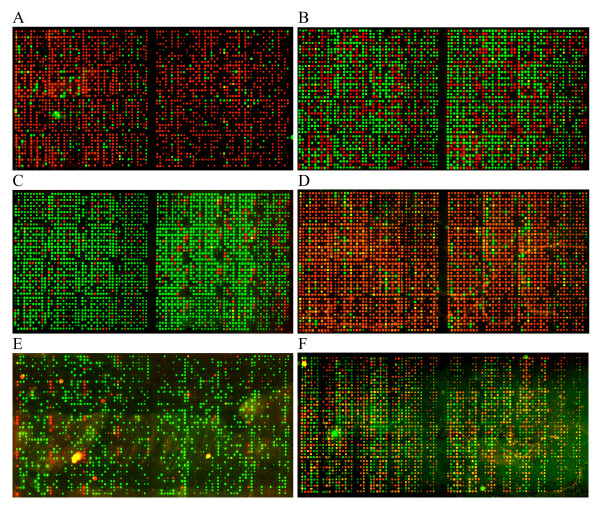
**Examples of TAM arrays for RBIP markers scored in the JIC *Pisum *Collection**. Each array scores a single retrotransposon insertion polymorphism in 3029 *Pisum *DNAs. Markers illustrated [19] are as follows: **A**, UniTpv; **B**, 281 × 44; **C**, 1794-2; **D**, 95 × 25; **E**, Birte-x34; **F**, 64 × 11. The arrays also contain 672 blank spots (no sample arrayed) 128 positive controls 9 DNA duplicates and 42 PCR negative controls (mock DNA preparation with no input plant sample).

The method used is described in detail in [17]. Briefly, PCR products were spotted directly from PCR plates without purification onto a streptavidin-coated microarray slide (made by us) which was pre-hybridised then hybridised with a mixture of two detector probes, carrying corresponding fluorescent labels (Cy3 or Cy5). The slides were washed to remove unbound probe and scanned using an ArrayWorx™ Biochip Reader. Representative images are shown in Figure 1 and the full set of images are available in Additional file 2 and from the Germinate pea database (Additional file 3) [22,23].

### Marker data acquisition and quality control

Collected pixel data for each microarray obtained from the array reader were converted into spot pixel intensities using the manufacturer's software and exported to excel spreadsheets. Spot signal intensities were corrected for local backgrounds (both Cy3 and Cy5) then corrected intensities for each spot were normalised against each other using the strongest reliable spot signals for each dye in each array. No-score cut-offs for weak spots were obtained by comparing background-corrected signals for each fluorochrome with visual inspection of the corresponding array image. Any corrected signals falling below the cut-off threshold (typically, fluorescence values less than twice the local background) were set to zero, giving final corrected signals for each fluorochrome for each spot. Final sample scores were obtained by calculating ratios between these two final signals for the corresponding spots. Typically, spots below the cut-off in both channels were given no-score, spots with Cy3/Cy5 ratios greater than 3 were scored as Cy3, scores with ratios between 0.3 and 3 were scored as mixed (Cy3 + Cy5) and scores with Cy3/Cy5 ratios below 0.3 were scored as Cy5. For some RBIP markers these scoring parameters were adjusted to fit obvious groupings of germplasm subsets in the plotted fluorescence intensities [see individual marker plotted scores in the Germinate database; [22,23] and in a few cases parallel gel-based scoring of the PCR products were used to 'train' the above scoring parameters (see below). Finally, Cy scores were converted to allele scores (unoccupied/occupied/mixed/no score), depending upon the fluorescent tag detectors used in the assay [17].

Preliminary inspection of the 80 marker arrays showed 29 markers of very good quality (e.g. Figure 1A-C). We also found 15 markers of very poor quality, for example some had missing data of around 50% or more (e.g. Figure 1E) or were virtually mono-allelic in the entire population (e.g. Additional file 2, marker 261 × 1). These markers were discarded, leaving 36 remaining markers to be scrutinised. Many of these showed large numbers of mixed fluorescence signals (e.g. Figure 1D, 1F). The plants used for these studies were all homozygous (see Introduction and *Plant Materials *and *DNA extraction *above) and high levels of mixed spots implied that more than one locus being sampled by the TAM assay [16,17,19]. Therefore, all 36 remaining markers were tested by agarose gel electrophoresis of the PCR products (RBIP amplicons are designed to show size polymorphism). 16 of these markers gave very good correlation between gel bands and corresponding spot fluorescence and were added to the 29 already accepted, resulting in 45 markers accepted for downstream data analysis (Table 1).

**Table 1:** Markers used in this study

Marker	Insert	Array Result	Used in this study?
45 × 8	*PDR1*	Many missing scores, Cy3/Cy5 false positive problem	No
45 × 15	*PDR1*	Good	Yes
45 × 20	*PDR1*	Very weak signals, Cy3/Cy5 false positive problem	No
45 × 29	*PDR1*	Very weak signals, Cy3/Cy5 false positive problem	No
45 × 31	*PDR1*	Slight false positive problem	Yes
45 × 33	*PDR1*	Slight Cy5 false positive problem	Yes
45 × 38	*PDR1*	Very low polymorphism, Cy5 false positive problem	No
64 × 11	*PDR1*	Many weak or mixed Cy3/Cy5 scores	No
64 × 14	*PDR1*	Rather low polymorphism	Yes
64 × 15	*PDR1*	Many weak or mixed Cy3/Cy5 scores, Cy5 false positive problem	No
64 × 29	*PDR1*	Very low polymorphism, Cy3 false positive problem	No
64 × 40	*PDR1*	Many weak or mixed Cy3/Cy5 scores, false positive problem	No
64 × 45	*PDR1*	Many mixed Cy3/Cy5 scores, Cy5 false positive problem	No
64 × 74	*PDR1*	Many mixed Cy3/Cy5 scores, false positive problem	No
64 × 76	*PDR1*	Rather low polymorphism	Yes
95 × 2	*PDR1*	Slight Cy5 false positive problem	Yes
95 × 19	*PDR1*	Good	Yes
95 × 25	*PDR1*	Slight Cy5 false positive problem	Yes
95 × 43	*PDR1*	Good	Yes
261 × 1	*PDR1*	No polymorphism	No
261 × 13	*PDR1*	Cy5 false positive problem, very low polymorphism	No
281 × 1	*PDR1*	Slight Cy3 false positive problem	Yes
281 × 5	*PDR1*	Rather low polymorphism	Yes
281 × 16	*PDR1*	Good	Yes
281 × 40	*PDR1*	Slight Cy5 false positive problem	Yes
281 × 44	*PDR1*	Good	Yes
399-3-6	*PDR1*	Cy3 false positive problem, very low polymorphism	No
399-14-9	*PDR1*	Good	Yes
399-80-46	*PDR1*	Slight Cy5 false positive problem	Yes
399-9x	*PDR1*	Slight Cy5 false positive problem	Yes
399 × 131	*PDR1*	Slight false positive problem	Yes
399 × 149	*PDR1*	Slight false positive problem	Yes
1006nr2	*PDR1*	Low polymorphism; Cy3 false positive problem	No
1006nr9	*PDR1*	Very poor image	No
1006nr13	*PDR1*	Good	Yes
1006nr27	*PDR1*	Good	Yes
1006nr32	*PDR1*	Weak signal; multiple spot colours	No
1006 × 6	*PDR1*	Slight Cy5 false positive problem	Yes
1006 × 19	*PDR1*	Low polymorphism; Cy5 false positives problem	No
1006 × 21	*PDR1*	Slight Cy3 false positive problem	Yes
1006 × 36	*PDR1*	Many signals weak or absent; Cy5 false positive problem	No
1006 × 50	*PDR1*	Some Cy5 background signal (Yellow spots)	Yes
1006 × 58	*PDR1*	Some Cy5 background signal (Yellow spots)	Yes
1794-1	*PDR1*	Good	Yes
1794-2	*PDR1*	Good	Yes
1794 × 7	*PDR1*	Slight Cy3 false positive problem	Yes
1794 × 9	*PDR1*	Very low polymorphism	No
1794 × 35	*PDR1*	Many signals absent; Cy5 background signal	No
2055nr1	*PDR1*	Good	Yes
2055nr16	*PDR1*	Low polymorphism, good otherwise	Yes
2055nr23	*PDR1*	Rather low polymorphism, good otherwise	Yes
2055nr51	*PDR1*	False positive problem, weak signals	No
2055nr53	*PDR1*	Slight Cy3 background signal	Yes
2055 × 10	*PDR1*	Many signals weak or absent; Cy5 false positive problem	No
2055 × 19	*PDR1*	Low polymorphism, weak signals, many mixed Cy3/Cy5 signals	No
2055 × 28	*PDR1*	Low polymorphism, weak signals, Cy3 false positive problem	No
2055 × 29	*PDR1*	Many signals weak or absent; Cy5 false positive problem	No
2055 × 36	*PDR1*	Cy3 false positive problem	No
2201CycL6	*PDR1*	Rather low polymorphism	Yes
2385 × 16	*PDR1*	Very low polymorphism, mixed Cy3/Cy5 signals, Cy3 false positive problem	No
2385 × 23	*PDR1*	Rather low polymorphism	Yes
2385 × 46	*PDR1*	Very weak signals, Cy5 false positive problem	No
2385 × 56	*PDR1*	Very weak signals, Cy3/Cy5 false positive problem	No
2385 × 64	*PDR1*	Rather low polymorphism	Yes
2539 × 7	*PDR1*	Some Cy5 contaminating signal	Yes
3150 × 11	*PDR1*	Almost no polymorphism	No
Birte-B1	*PDR1*	Good	Yes
Birte-x5	*PDR1*	Very low polymorphism, many very weak signals	No
Birte-x16	*PDR1*	Good	Yes
Birte-x28	*PDR1*	Some weak or mixed Cy3/Cy5 scores	Yes
Birte-x34	*PDR1*	Good	Yes
MKRBIP2	*PDR1*	Slight Cy5 contamination problem	Yes
MKRBIP3	*PDR1*	Many missing scores	No
MKRBIP7	*PDR1*	Good	Yes
Cycl1074-L12	*Cyclops*	Some Cy5 contaminating signal	Yes
Cycl1074-L29	*Cyclops*	Good	Yes
Cycl711-L12	*Cyclops*	Many missing scores, many mixed Cy3/Cy5 scores	No
UniTpv	*Tpv1*	Good	Yes
DBAP-261 × 1	Indel	Very Low polymorphism	No
DBAP-2055nr53	Indel	Very Low polymorphism	No

The origin accession for each RBIP marker is given typically by the first number in the RBIP designation (*ie *marker 45 × 8 derived from JI45). Exceptions to this rule are MKRBIPs (which derived from mapping populations between accessions JI15, JI281 and JI399), Birte markers which derived from JI1068 [*cv Birte*] and *Uni-Tpv *which derived from cultivar Therese (not in the JI Collection).

Scoring reproducibility between arrays was explored for four randomly selected markers (Birte-x16, 45 × 31, 2055nr1 and 281 × 16, data not shown). Reproducibility of Cy3 and Cy5 scores for the same set of PCRs arrayed onto different slides was high (89% and 98% respectively) and scoring reproducibility between different PCRs using the same samples was lower (64% and 85% respectively). The great majority of these discrepancies between scores corresponded to no scores in one of the arrays (38%; SD 9% and 17% SD 5% respectively). In many cases this corresponded with poor spotting efficiency of particular spotting pins (Additional file 2). This interpretation was supported by comparisons between gel and array scores (161 scores per marker) for each of 7 other accepted markers and all 3029 scores for Birte-x16. These showed false negative rates (array no-score/gel score) of between 0 and 0.24 per marker and false positive rates (array score/gel no-score) of between between 0 and 0.12 per marker for the 7 markers. For Birte-x16 95% of the TAM no-score samples gave scores by gel, confirming the above interpretation. Therefore, all the above discrepancies between scores and no-scores were resolved in favour of scores for all repeated arrays. Discrepancies between occupied scores in one array and unoccupied in another was extremely low (average 0.13%; SD 0.08%). Lastly, the reproducibility of mixed scores ('yellow spots') between arrays was poor (25% and 22% within the same PCRs and between different PCRs respectively).

The inclusion of 9 duplicated samples (see above) allowed us to test scoring reproducibility further. In general the score data for these 9 duplicate pairs were very similar to the above data for repeat PCRs, with no-scores for a single sample casuing almost all discrepancies (data not shown).

### Data storage and visualization

All marker allele scores, together with original array images, passport data and other descriptive data for the *Pisum *accessions in this study were stored in an open-access relational database built using the Germinate Open Source Platform [22,23]. All marker data in this study can be freely browsed at the Germinate-Pea database [23] or obtained as an excel file from AJF. The Germinate database (Additional file 3) [23] was re-written in MySQL to remove the limitations imposed by reliance on PostgreSQL and to cater for the larger number of end users working in this area who are familiar with MySQL.

Germinate-Pea is equipped with a Perl web-interface and tools and administration tools written in Perl to aid users in configuring and maintaining data and Germinate's installation features. The interface allows browsing and string searching of all data (for example, to find information on a single accession, linked where applicable to geographical map information; Additional file 4A). A Group facility allows storable, password-protected grouping of analysis outputs and groups can be merged, subtracted, overlapped *etc*. Data subsets (accession, marker or marker × accession) are retrievable and any data (sub)set is exportable as tab-delimited text and/or kml file for downstream analysis or visualization (*e.g*. for spreadsheet or Google Earth respectively). Original array images and corresponding pseudoimages of inferred marker scores are visualised (Additional file 4B). The parent Germinate platform is currently in version 2.3 and is downloadable at the Germinate website [24].

### Phylogenetic and genetic diversity analysis

All final marker score data were combined into an excel spreadsheet. 'Yellow spots' (samples scoring both occupied and unoccupied for a given marker) were treated as missing data. Analyses were performed using the DARwin5 package [25,6] and STRUCTURE [27,28]. Structure (v2.1) accepted the entire data set without further assumptions about the nature of missing data. Individuals were considered to be homozygous for all loci as the DNA preps were from a single leaflet of a single inbred accession. Structure parameters discussed in the text follow the definitions of Pritchard *et al*. [27] and Evanno *et al*. [29] thus: **K **= The number of proposed ancestral populations. K is user defined and we have analysed values between 2 and 21. **Q **= The representation of a given ancestral population in an accession. Structure attempts to describe the representation of more remote ancestors, that themselves have various degrees of relationship, the values of Q can range widely. Note that two individuals with identical values of Q for a series of proposed ancestors, need not necessarily carry an identical set of alleles.

For Structure the default parameter set was used throughout, using the admixture model, but the effect of run length was investigated. Most runs comprised 10,000 Markov Chain Monte Carlo (MCMC) runs after a 'burn-in' of 10,000. These runs were replicated ten times for each K and each data set or subset analysed and were compared to runs comprising 800,000 MCMC runs after a 'burn-in' of 200,000. The replicated shorter runs gave similar results so these were used because their duration was substantially shorter. Values of K to explore were chosen according to [29] calculated in Excel tables. Correlation analysis of the assignments of Q between different populations for accessions in different runs was performed using GenStat [30]. Genetic distances between Structure Sub-Groups were calculated according to Nei's method using 'Genedist' in the Phylip package [31,32] and the resulting tree was plotted using DARwin5 [26]. Factorial analysis of the data was calculated by a simple match of shared alleles for all pairs of accessions. The proportion of shared alleleles for each accession pair was calculated using a simple BASIC program (available on request).

### Robustness of Structure descriptions

A Structure description can be considered robust if it is obtained from multiple independent runs of the program. In practice independent runs for our large data sets were never identical, but the assignments of ancestry were broadly similar for 'well correlated' runs. We assessed this robustness by determining the correlation between assignments of Q for each run. For a structure run 'S', with K populations, we can call this value Q_SK_.

For K = 2, in a given run (G) an individual accession has two values of Q: Q_G1 _and Q_G2_.

We therefore have four possible correlations between the population assignments for all accessions: R_QG1QG1_, R_QG2QG2_, R_QG1QG2 _and R_QG2QG1_. As any value is identical to itself R_QG1QG1 _= 1 and R_QG2QG2 _= 1, and as Q_G1 _+ Q_G2 _= 1 it follows that R_QG1QG2 _= -1 and R_QG2QG1 _= -1. The sum of these correlations is therefore zero.

If we compared two different K = 2 runs (A and B) we could decide whether the assigned parental populations were or were not the same by looking at the correlations R_QA1QB1_, R_QA1QB2_, R_QA2QB1_, R_QA2QB2_. If the assignments were identical we would again have two values of 1 and two of -1. Comparing the sums is not informative, because they are zero, but comparing the sums of the positive correlations immediately identifies corresponding proposed parental populations. High positive correlations suggest that the proposed parental populations are very similar.

Thus if a pair of Structure runs has a high sum of positive correlations between proposed partitioning of parental populations then they are proposing essentially the same parental populations and the same partitioning of their variation among the sampled accessions. Thus the sum of their positive correlations can be regarded as a measure of robustness, and in general can be compared for different values of K if divided by the sum of the two K values being compared. This can hold for runs with different values of K, where a single population in the lower K value may be partitioned simply into several populations at higher K. Note that two runs may propose one or a few parental populations that are similar and others that are different. This means that although a run may not propose all the same parental populations it may propose a subset in a consistent manner.

If the founder populations had differing degrees of relatedness some may be 'found' more easily by Structure than others, and closely related populations may be compounded in different permutations by different Structure runs. This suggests that a hierarchical approach to partitioning populations may be fruitful because the problem of identifying parental populations is simplified if we take a restricted germplasm set.

### Neighbor joining tree analysis

Structure analyses assigned the ancestry of individuals among three population Groups. Individuals with at least 50% of their genome assigned to Group 1, 2 or 3 were further assigned by subsequent Structure analysis to six, two or six Sub-Groups respectively. The relationship between these populations of accessions was then described by the allele frequency between the Sub-Groups. For each Sub-Group the frequency of empty sites was counted and represented as a fraction of empty plus occupied sites. A matrix of this fraction, for each marker and assigned Sub-Group, was then used to generate a distance matrix between the Sub-Groups corresponding to a population using the GENEDIST program in the Phylip package (http://evolution.genetics.washington.edu/phylip.html, [30,31]), assuming one allele omitted and using Nei's genetic distance. The resulting distance matrix was used to construct a neighbor joining tree using the Neighbor program of the same package. The resulting tree therefore represents the relationship between the collections of accessions proposed by Structure, assuming that these accession sets were real populations. Note that the relationship between these sets of accessions is not the same as the relationship between the proposed ancestral populations (as described in the Structure output files), but is a description of this way of partitioning the existing germplasm collection.

### Cluster analysis

Multivariate statistical approaches can condense the description of the relationships between multiple individuals. We used the DARwin5 program [24,25] to undertake a factorial analysis of the marker data for the whole set of accessions. This represents the total variance of the inter-accession distances in 3028 dimensions, and the first two dimensions (comprising 5.32% and 2.99% of the total variation respectively) were used to display the relationship between individuals.

## Results

### RBIP marker scoring of the John Innes *Pisum *Collection

The objective of this study was to determine the genetic structure of a large, complete and diverse *Pisum *germplasm collection. The John Innes *Pisum *Collection, is widely used as a source of pea germplasm with a particularly good representation. At the time of sampling in 2003, the collection comprised wild (407), landrace (564), breeders' lines (61), cultivated (1021) and mutation stocks and research lines (835) accessions. A good body of the wild and landrace accessions had associated geographical data. DNAs were prepared from 2776 of the 2888 accessions sampled, together with 208 private lines, 36 lines not yet included in the collection and 9 duplicates, corresponding to 3029 samples in total. The marker technology used was the TAM approach [16,17] which allows high throughput codominant scoring of polymorphic transposon insertions by microarray or gel electrophoresis. 78 RBIP markers [19], plus 2 adjacent short indel markers were available to us. These markers were all scored in the 3029 samples, representative examples of TAM arrays are shown in Figure 1. Careful scrutiny of these arrays and comparison with parallel gel-based assays of subsets of the PCR products resulted in the rejection of 35 of these markers (see Materials and Methods), leaving 45 useful marker score sets carried forward for the study. The 80 array images are all shown in Additional file 2 and the complete molecular data set and associated images for the 45 markers and all available passport and descriptive data for the accessions are available at the Germinate Pea database (Materials and Methods; Additional file 3) [23].

### Bayesian analysis of population structure for the JI Pisum Collection

The marker data set comprised 40,790 alleles called as occupied sites (insertion present)), 65,566 unoccupied sites (insertion absent) and 29,949 missing data points (no detected PCR product or both occupied and unoccupied sites together). We explored the diversity of this data set using the program Structure [27,28,33]. Structure uses a Bayesian approach to allocate proportions (Q) of K presumed ancestral populations contributing to each sample (see Materials and Methods). Individuals may be assigned as the descendant of one population or having an admixture of two or more presumed ancestral populations. K is an important parameter which shapes the subsequent inferred sub-division of genetic structure, although it is a summary and convenience rather than a strictly defined hypothesis about the origin of a given set of individuals. In practice multiple K values are explored and the value accepted is usually that which gives the maximum value of the likelihood of the data, ln(pr(Data|K)).

**Figure 2:**
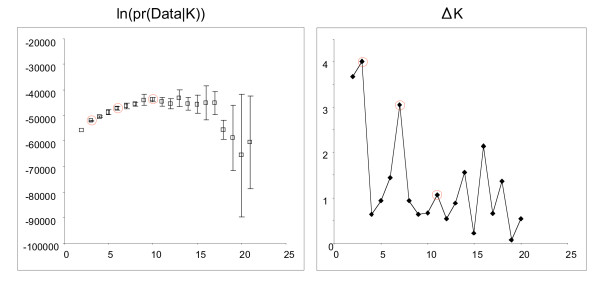
**Exploration of K value for Structure analysis of the JIC *Pisum *Collection**. A. Mean values of the log likelihood of the data given K is plotted against K for 10 simulations with a burn-in of 10,000 runs followed by 10,000 MCMC runs. The error bars show the standard deviations of the mean values. B. Estimates of the rate of change of the slope of the log likelihood curve (ΔK) calculated according to [29] are plotted against K. K values of 3, 7 and 11 giving robust ΔK maxima were investigated further.

**Figure 3:**
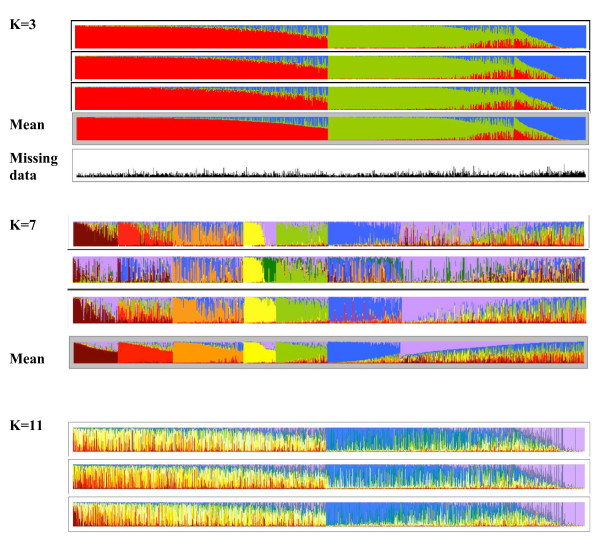
**Structure analysis of the JIC *Pisum *Collection: Comparing different K values**. The results of three Structure runs for K values of 3, 7 and 11 are shown, together with the means for 10 K = 3 runs and the 3 most reproducible K = 7 runs. For each run the fractional inferred ancestry of the 3029 individuals is plotted as a histogram, with each ancestral population colour-coded. Accessions are assigned to Sub-Group N if their average representation (Q_N_) for that Sub-Group ≥ 0.5. Accessions in Groups 1 and 2 are ordered by decreasing Q and for group 3 by increasing Q. Admixtured accessions, where Q_1_+Q_2 _≥ 0.5, are placed between groups 2 and 3, ordered by increasing Q_3_. For K = 7 the accessions are ordered within Sub-Groups in a similar way. For K = 11 there was no consistent assignment between Structure runs of accessions to groups; therefore the accession order is the same as for K = 3 (see also Additional file 4). At the bottom of the K = 3 figure the fraction of missing data per accession is plotted as a black vertical bar.

In this study we explored K between 2 and 21 inclusive, with ten independent assessments of the log likelihood of the data made for each K value. For our analysis the log likelihood does not reach a clear maximum (Figure 2A). This phenomenon has been seen in other studies [*e.g*. [34]] and reflects heterogeneity between simulations in the distribution of ancestry proportions from the progenitor populations. One proposed solution to this problem is to use maxima in the second derivative (ΔK) of the relationship between K and the log-likelihood as an appropriate way of selecting K, thus effectively looking for 'shoulder' in the first plot (Figure 2B) [29]. Several possible values of K were indicated by this method but the maxima tended to be less reliable between runs at higher K values. Simulations at K = 3, 7 and 11 in Figure 2a were examined for internal consistency both graphically and by correlation analysis. Examples of this are shown in Figure 3 and all the simulations and correlations for these K values are shown in Supplementary Figures 3 and 4. While there are some reasonably robust Sub-Groups identified within the K = 7 Structure runs, the K = 3 simulations are the most consistent by far between runs (Figure 3, Additional file 5) and we propose that this value represents the most robust division for the data set and the start point for exploring the diversity of the JIC *Pisum *germplasm. The grouping of the data into essentially these three populations was also seen when using 27 of these marker loci (data not shown), suggesting that the relationships this implies are a robust feature of the data set.

**Figure 4:**
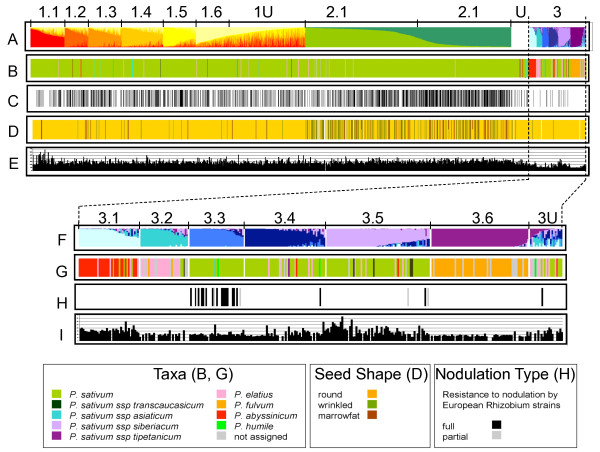
**Sub-structuring of K = 3 Structure groups and relationship to taxonomy and domestication traits**. A: Nested Structure analysis for the 3 groups identified by the average of 10 independent K = 3 Structure simulations for the JIC germplasm (Figure 3) with K values of 6, 2 and 6 assigned to Groups 1-3 respectively. Accessions have been placed in order according to their average representation within the subgroups. B: Species assignments according to the key below. C: Cultivar assignments (each black bar is a cultivar). D: Distributions of important seed traits, indicated by colour according to the key below. Wrinkled includes, but is not limited to, *rugosus *mutants. E: Seed weights, F: Blow-up of Group 3 sub structuring (average of the 7 best correlated runs). G: Species assignments according to the key below. H: Identification of cv Afghanistan types according to [33]. Black bars are 'resistant' types (*sym2*) while grey bars are accessions show 'partial' resistance to nodulation by European *Rhizobium *strains [33]. I: I: Seed weights.

The relationships between a single pair of K = 3 and K = 7 Sub-Groups are shown for illustration in Additional file 5. The K = 7 grouping is less reproducible than that for K = 3 so the former data are ordered by the average of the 3 most consistent runs and there are many admixed accessions in the former run with no dominant progenitor and consequently no Sub-Group assignation (top sectors of 3C). The K = 3 Group 1 (red) corresponds to three K = 7 Groups plus the K = 7 unassigned subset (top of the Figure), The second K = 3 Group (green) corresponds quite closely to two other K = 7 Groups and the third K = 3 Group (blue) corresponds closely to a single K = 7 Group. It therefore seems that higher K values provide some credible sub-partitioning of the K = 3 groupings but at the cost of reduced reproducibility.

The distribution of missing data relative to the K = 3 Structure Groups is shown in Figure 3. The average fraction of missing data in each K = 3 group is 0.206 ± 0.003 for Group 1 (red), 0.219 ± 0.004 for Group 2 (green), 0.279 ± 0.007 for Group 3 (blue) and 0.246 ± 0.016 for the admixed accessions. The slight excess of missing data for Group 3 is significant (p < 0.001). This is a predominantly wild Group which contains a large fraction of the total genetic diversity of the entire population (see below) and we suggest that the elevated fraction of missing data is mainly due to PCR failure at mutated priming sites.

### Investigation of sub-structuring of *Pisum *diversity

The results of Structure analysis using higher K values described above suggested extra sub-structuring of the diversity above that of K = 3. Therefore, each of the three K = 3 sample sets were treated individually to further Structure analysis (Figure 4). Analysis of the relationships between K and ΔK for these data sets (data not shown) suggested that K = 6 would be a helpful descriptors for Group 1, K = 2 was indicated for Group 2 and K = 6 for Group 3 (data not shown). Sub-groups generated by Structure runs using these K values are shown in Figure 4A and 4F, together with taxonomic assignment (Figure 4B, 4G; Additional file 6), and domestication-related trait parameters of accessions (Figure 4C-E; 4H-I).

Group 1 is dominated by *P sativum *(Figure 4B) and is comprised of a mixture of landraces and cultivars (Figure 4B-C), the great majority of which have round seed phenotypes (orange in Figure 4D). Sub-Group 1.1 contains a high proportion of large seed lines (Figure 4E) but there is little obvious partitioning of seed shape or size between the other Group 1 Sub-Groups. Group 2 is dominated by *P. sativum *cultivars (Figure 4B-C) and contains the bulk of the wrinkled seeded types (Figure 4D; note these include, but are not limited to, mutants at the various *rugosus *loci), with again no obvious partitioning by wrinkled phenotype between to the two Sub-Groups. There is a larger proportion of cultivars in Sub-Group 2.2 relative to 2.1 (χ^2 ^= 32, p < 0.001) and this is reflected in 6% greater mean seed size for Sub-Group 2.2 relative to Sub-Group 2.1 (Figure 4E, data not shown). In summary, there is some credible segregation for taxonomy, cultivar and seed parameters among some of the Sub-Groups of Groups 1 and 2 but this is not dramatic.

In contrast to the first two Groups, Group 3 displays a considerable amount of sub-structuring, with regard to both taxonomy and phenotypic traits (Figure 4F-I; Additional file 6). Sub-Group 3.1 contains almost all *P. abyssinicum *accessions, Sub-Group 3.2 contains about one third of the *P. elatius *lines and Sub-Group 3.6 contains 96% of the *P. fulvum *accessions (Figure 4G). Sub-Groups 3.3-3.5 are mostly *P. sativum *landraces (Figure 4C, 4G) and 3.3 is characterised by the presence of the 'c.v. Afghanistan' types [35] that carry the *sym2 *allele conferring resistance to nodulation by European *Rhizobium *strains (Figure 4H). Lastly, most of Group 3 has small seed size, with the notable exceptions of Sub-Group 3.1 (cultivated *P. abyssinicum*) and a fraction of Sub-Group 3.5 derived mainly from Afghanistan and India (data not shown). We will show below that much of this sub-structuring of Group 3 is also reflected at the geographical level.

**Figure 5:**
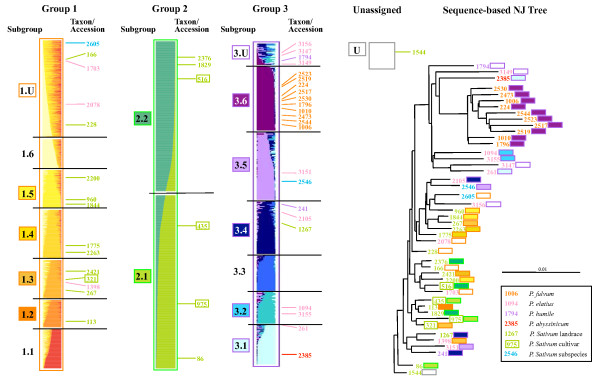
**Relationships between RBIP-based Structure Sub-Grouping and sequence-based tree structure**. Accessions assigned to Sub-groups of the three K = 3 Groups (see Figure 4) are shown on a previous neighbour joining tree based on intron sequence [adapted from [17]]. Subgroup assignments are indicated by box fill colours, Group assignments are shown by box outline colours and taxonomic (species/sub-species) assignments by colour coded text, using colours from Figure 4. 'Unassigned' Subgroups U, 1.U and 3.U (white boxes) are comprised of accessions with no founder genotypic contribution of 50% or more in the corresponding Structure run. Subgroups not represented in the tree (Subgroups 1.1, 1.6 and 3.3) are unboxed.

The above data suggest that the Sub-Groupings identified by Structure for Groups 1 and 2 are supported to some extent by prior phenotypic or taxonomic data and the sub-structuring of the wild-dominated *Pisum *Group 3 makes very good sense in these contexts. To assess to what degree these Groups and Sub-Groups are supported by previous analysis, we investigated the concordance between Structure grouping and the corresponding gene sequence-based tree structure for a set of 45 well studied JIC Collection accessions [10] (Figure 5). 15 of these accessions belong to Group 1, 6 to Group 2, 23 to Group 3 and 1 is unassigned. All Group 1 and 2 accessions are located in the bottom two-thirds of the tree and the internal branch lengths in this sector are typically short; these branching patterns are poorly supported [10]. Five of the six Group 2 accessions are confined to two weakly supported clades towards the bottom of the tree, with the sixth nearby. Somewhat surprisingly, six Group 3 samples, which constitute all of the accessions sampled here from Sub-Groups 3.4 and 3.5, plus one unassigned accession, occupy two weakly supported and quite distantly separated clades in the lower region of the tree. There is no obvious correlation between the 3.4/3.5 division and assignment to these clades. Also, each clade contains one or two samples from Sub-Group1. The rest of Group 3 (17 out of 23 accessions) constitutes the entirety of the two most strongly supported clades in the tree, one nested within the other, towards the top of the tree. The nested clade comprises all 10 *P. fulvum *samples and all belong to Sub-Group 3.6. Additionally, all 4 samples from Sub-groups 3.1 and 3.2 reside in the outer clade. We conclude from this comparison that; i) Much of the Sub-structuring identified in Group 3 is broadly supported by sequence-based diversity assessment of a small representative subset of the samples but Sub-Groups 3.4 and 3.5 are distributed differently by the two diversity approaches. ii) There is little agreement apparent between the Sub-Grouping of Groups 1 and 2 and the corresponding sequence-derived tree structure.

### Distance-based estimations of the relationships among Structure Sub-Groups

**Figure 6:**
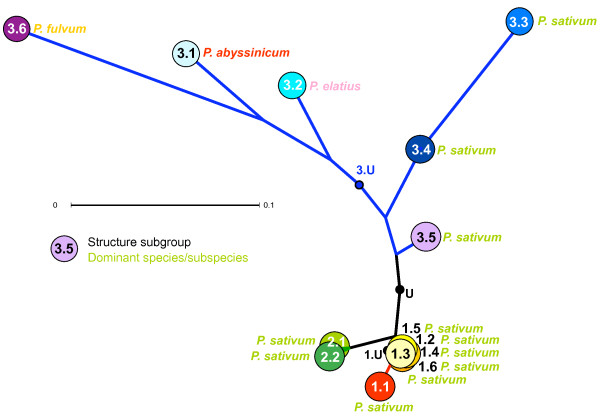
**Distance-based estimations for Structure Sub-Groups**. A neighbor joining tree of the genetic distance between Sub-Groups was calculated from the combined allele frequencies per Sub-Group, using Nei's method [40]. Structure Sub-groups are colour coded as in Figure 4 and predominant species in each Sub-Group (Additional file 2) are indicated by coloured text using the same species-specific colours as Figure 4.

The above data show that *Pisum *genetic diversity is well described by three major groupings, which very broadly correspond to *P. sativum *landraces, *P. sativum *cultivars and wild samples respectively, with various Sub-Groupings of these supported to a greater or lesser extent by taxonomy, trait data or corresponding sequence-based tree structure. This description gives no direct idea of the genetic distances between these groupings or their phylogenetic relationships. It is possible to obtain this by comparing the marker allele frequencies between Sub-Groups, thus treating each Sub-Group as a genetic unit. The deduced tree from this analysis is shown in Figure 6. Most of the genetic divergence between these sub-groups of accessions is within Structure Group 3 (blue branches), and the sub-groups within Groups 1 and 2 are very poorly resolved. This suggests that relatively little marker information is available to define the sub-grouping within Groups 1 and 2, consistent with the weaker support for these subgroups described above.

Interestingly, Sub-Groups 3.4 and 3.5 are the nearest neighbours to Groups 1 and 2 and these also cluster in the sequence-based tree (Figure 5). Both 3.4 and 3.5 are dominated by non-adapted *P. sativum *(Additional file 2). We will return to these points later when discussing models of *P. sativum *domestication. Sub-Group 3.3, corresponding to the Afghan class of *P. sativum*, is an outlier to Sub-Group 3.4. Finally, Sub-Groups 3.2 and 3.6, which are dominated respectively by the wild species *P. elatius *and *P. fulvum *(Additional file 6), are remote from *P. sativum*, with the *P. abyssinicum *dominated Sub-Group 3.1 roughly equidistant between them, in agreement with previously derived relationships based upon other marker types [6,10].

**Figure 7:**
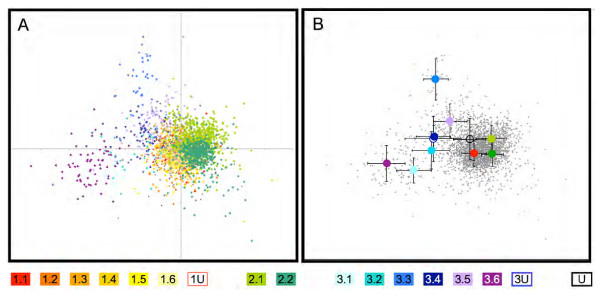
**Multifactorial analysis of *Pisum *diversity**. For the entire data set the fraction of shared alleles for all pair-wise combinations of samples is analysed by multidimensional scaling. The output for the first two dimensions (explaining 5.32% and 2.99% of the variation are shown (see text). A: All points are plotted with each sample is colour-coded according to its corresponding sub-group membership. B: The mean of all values for each sub group, together with standard deviations are plotted. Sub Groups are colour coded as shown below. The subgroups of Group 1 lie so close together that they are not individually identified but collectively represented in red. All points are plotted in grey and the axes are removed for clarity.

We also used multi-factorial analysis of the marker data set to analyse the diversity of the *Pisum *germplasm (Figure 7). The results in general support the above findings for the relationships between the Sub-Groups and they also show both the diversity within Sub-Groups and the extent to which the Sub-Groups overlap. The area occupied by a germplasm sub-set relative to the total area occupied in the multi-factorial plot gives a measure of its contribution to the overall diversity of the complete set. This can be expressed as the product of the standard deviation of the X and Y values for the individuals in the sub-set. For Groups 1-3 these values correspond to 12.5%, 15.6% and 71.9% of the total respectively (Figure 7; data not shown). Thus, Group 3 represents most of the variation of *Pisum *as a whole, assuming that the JI Collection is a good representation of the genus, and its Sub-Groups are quite well resolved, particularly Sub-Group 3.3, with 3.1 and 3.6 also quite distinct. These data are consistent with the Structure-based tree (Figure 6) and prior analyses [6]. Group 2 is partially resolved into its two components but Group 1 is very poorly resolved and forms a large cluster. We conclude that the Group 3 Sub-Groups largely represent distinct germplasm by multi-factorial analysis, the two Sub-Groups of Group 2 are partially resolved and Group 1 is predominantly homogeneous by this approach.

### Relationship between Structure Sub-Group and country of origin

**Figure 8:**
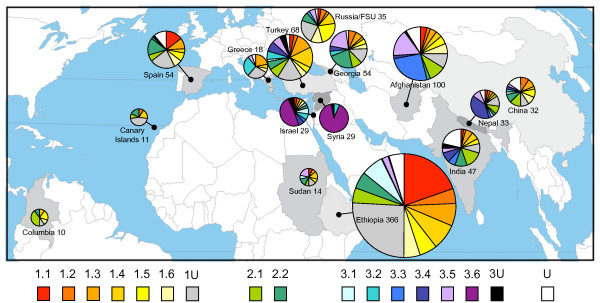
**Geographical Distribution of Structure Sub-Groups of the JIC *Pisom *Germplasm**. Data are shown for all donor countries contributing 10 or more accessions in the JIC *Pisum *Collection. Each piechart refers to a single country, the area of the piechart reflects the number of accessions derived from that country and the corresponding compositions by Structure Sub-Group (colour-coded as in Figures 4-6) are indicated. The map is derived from [41].

There is geographical information available for roughly a third of the accessions in the JIC collection, comprising the majority of the wild and landrace material. Much of this information defines a country of origin while exact collection site location is lacking. Nevertheless, this allowed us to investigate distribution by country for the Sub-Groups in this study (Figure 8). Several clear correlations between Sub-Group and country of origin are apparent. Firstly, almost all of Sub-Group 1.1 is assigned to Ethiopia and Spain, with a significant amount of Dutch material also (data not shown). This is the only Sub-Group of Group 1 that can be resolved by genetic distance (Figure 6) and the other Group 1 Sub-Groups show little bias for country of origin. Second, Sub-Group 3.6 (the *P. fulvum *Sub-Group) is largely confined to Israel and Syria, as expected from the known geographic origin for the species. Similarly, the *P. abyssinicum *Sub-Group 3.1 mainly derives from Ethiopia and the *P. elatius *Sub-Group 3.2 is distributed across the Eastern Mediterranean, as expected. The *P. sativum *Sub-Groups 3.3-3.5 also show marked concentration to individual countries or regions, namely Afghanistan, Nepal and South Central Asia respectively. Both Sub-Groups of Group 2 (*P. sativum *cultivars) are poorly represented in the country assignments and show little preference for country of origin. We deduce from these data that the Sub-Groups which are clearly resolved by marker analysis also show clear bias in region of origin, whereas poorly resolved Sub-Groups do not.

## Discussion

The main goal of this study was to genotype a large and genetically diverse *Pisum *germplasm collection and explore the diversity and evolution of *Pisum*. We chose the JIC *Pisum *germplasm collection because it is arguably the most complete *Pisum *collection worldwide, with a particularly good representation of wild and landrace material. Our analysis shows that the large majority of the genetic diversity of *Pisum *resides in these non-cultivar accessions (Figure 6). It is important to note that this higher diversity is not predominantly due to 'wild' alleles which are absent from cultivated genetic material [2]. In fact, only 13% of SSAP molecular markers from a diversity collection comprising 10 *P. fulvum *12 *P. elatius *and 5 *P. abyssinicum *JIC accessions are absent from 23 diverse *P. sativum *lines [[6]; Ellis *et al*, unpublished]. Thus, introgression across the species and sub-species in the *Pisum *species group has distributed alleles widely across the genus and it is the frequencies of these alleles in the different sub-populations, more than the presence of lineage-specific alleles, which are the major factor in *Pisum *diversity.

*Pisum *has relatively poor genomic resources at present and lacks the thousands of gene-linked high throughput SNP markers that are available for other cultivated plant species [36,37]. The RBIP markers chosen for this study are particularly useful for assessing genome diversity and evolution because they are biallelic, codominant and irreversible [15,19,20], but they are difficult to obtain in large quantities from plants with large genomes, because most retrotransposon insertions reside in other repetitious DNA hence the 'unoccupied' site may be present at many loci [17,19]. Furthermore, all PCR-based marker surveys at the genus level suffer with mutation at priming sites, leading to data loss. Lastly, retrotransposons are a more mutable genomic fraction than non-mobile DNA and individual insertions may be at extremely low allele frequencies, either because they have arisen relatively recently or have been nearly lost from the population through segregation [19]. Collectively, these shortcomings have lead to a high attrition rate (44%) in our candidate RBIP markers (Table 1). Nevertheless, the 45 markers scored here in 3029 samples comprise a large data set that has allowed us to explore the genetic diversity of *Pisum *to a wider extent than has been attempted previously.

### Analysis of *Pisum *genetic diversity using the Structure program

We chose Structure as the main tool for interpreting these marker data because it can handle very large sample numbers, the derived Q plots give very clear visual representation of the partitioning of genotype classes within the germplasm and it tolerates missing data without the need for assumptions about its causes. Our difficulty in obtaining a clear preferred K value has been seen frequently before [*e.g*. [28,34]]. We have found that increasing K values above 6 yields progressively less credible groups for this data set, even though likelihood values are still increasing slightly (Figure 2). This conclusion is consistent with that of Pritchard & Wen [28] that higher K values exaggerate population structure. In our opinion the approach of Evanno *et al *[29], which basically looks for 'shoulders' in the slope of K plots is useful, but care needs to be taken interpreting the ΔK peaks at higher K values (Figure 2B) because these are sensitive to the variance in the estimates of the log-likelihood values at K+1.

Because of the difficulties in obtaining a clear K value we have used a hierarchical approach, starting with a low, very robust value (K = 3), which reflects major divisions in the germplasm, namely (mainly) landrace, cultivar and wild respectively, then searching for internal structuring within these groups (see *Robustness of Structure descriptions *in Materials and Mathods). The rationale for this is in part convenience and in part biological. All *Pisum *had a single common ancestor (K = 1) and is represented in this study by 3020 genotypes (K = 3020), which are known to be distributed into some major subgroups (*P. fulvum *for example) or distinct breeding pools separated by time, geography, or cultivar type. These various historical processes occurred at different times and places and may represent quantitatively different numbers of individuals or proportions of total variation. Therefore, the number of 'ancestral populations' described by Structure is expected to differ over time, in both number and robustness, so no single value of K can describe the whole history of *Pisum*.

The relative success of our hierarchical Structure approach has varied greatly depending upon the Group analysed. The landrace-dominated Group 1 contains only one single well-resolved Sub-Group, namely 1.1 (Figure 6), which is also clearly seen when higher K values are used (Additional file 7), together with five other far less credible Sub-Groups. Group 2, which is mainly comprised of cultivar germplasm, comprises two poorly resolved Sub-Groups, suggesting little sub-structuring within the cultivated *Pisum *gene pool. None of these sub-groupings is well supported by parallel sequence-based distance tree analysis (Figure 5), though unfortunately the single example which might have been (Sub-Group 1.1) has no representatives in this other study. This is consistent with SNP data for barley cultivars, which cannot clearly resolve divisions beyond winter-spring growth habit (data not shown). Nevertheless subgroup 2.1 contains identified cultivars at a proportion expected from their distribution in the germplasm as a whole, while subgroup 2.2 has a significant excess of identified cultivars (χ^2 ^= 32, p < 0.001)

The power of our nested Structure approach becomes clear when the sub-structuring of Group 3, which mainly contains wild material, is explored and deep divisions between robust Sub-Groups are revealed (Figure 4). Three of these Sub-Groups correspond largely to well-defined *Pisum *species or sub-species (3.1 ≈ *P. abyssinicum*, 3.2 ≈ *P. elatius *and 3.6 ≈ *P. fulvum*). While it is unsurprising that any method for estimating genetic diversity reveals deep divisions at this phylogenetic level, our results highlight the advantage of this hierarchical Structure approach, because the K = 3 and K = 7 Structure runs did not dissect out these groupings consistently (Figure 3). It thus seems that a large excess of one germplasm type can confuse analysis by Structure. Furthermore, the other three Sub-Groups correspond to clear germplasm sets, each of which shares either phenotypic properties (Sub-Group 3.3; Figure 4H) or geographical range (Sub-Groups 3.3-3.5; Figure 8). Somewhat surprisingly, only Sub-Group 3.6 (*P. fulvum*) has unequivocal support from the DNA sequence-based distance analysis of a small germplasm subset of the JI Collection (Figure 5) but conversely, Structure has resolved credible exotic germplasm subsets (Sub-Groups 3.4 and 3.5) which appear by distance tree analysis to be closer to landrace *P. sativum *than they are to the wilder germplasm. We conclude that a hierarchical Structure approach is a valuable way of dissecting out complex sub-structuring in germplasm sets which can complement distance-based approaches by revealing extra diversity information.

The relationship tree derived from comparing allele frequencies across subgroups (Figure 5) enriches the Structure-derived conclusions by adding a distance dimension to the diversity revealed between Sub-Groups. However, this does not tell us about the differences between individuals within and between sub- groups. This is illustrated by a multi-factorial analysis (Figure 7), which shows that there are differences between individuals similar in scale to the differences between Sub-Groups. This pattern is consistent with the suggestion that *Pisum *as a whole is a single genetic entity with varying degrees of sub-division. The first and second dimensions of this plot shown in Figure 7 describe small components of the total variation (5.32% and 2.99% respectively), but this is more than 100-fold greater than the average expectation for the 3028 dimensions overall. Thus Structure appears to be identifying components of the genetic differentiation that are hard to determine by multivariate statistical approaches, but which nevertheless correspond to recognisable and coherent subsets of the gene pool.

Figure 6 and Figure 7 together show clearly that Groups 1 and 2 together represent a confined subset of the total diversity of *Pisum*. The cultivated types are a major sub-sample of these Groups. Thus, there has been a bottleneck associated with their derivation but this has not been severe compared to other species such as wheat and Brassica. Nonetheless, this emphasises the contrast that can exist between genetic diversity and representation in a germplasm collection - like almost all crop plant germplasm collections, the JIC collection is mostly made up of cultivated germplasm, while the smaller number of non-cultivated accessions represent a disproportionately large amount of the diversity. This is not surprising as collections often function as reference points for specific cohorts of stocks such as mapping populations or released cultivars from specific countries.

### A model for *Pisum *domestication

The correlations observed here between marker-based genetic diversity, domestication-related phenotypic traits and geographical origin reveals new insights into the origin and evolution of the genus *Pisum*. At the deepest level are the two unambiguous wild species *P. fulvum *and *P. elatius*. The former is well resolved by tree drawing methods, Structure analysis, and its limited geographical distribution (Figures 5-8) but *P. elatius *in contrast is present in multiple Sub-Groups, particularly 3.2, but also 3.1, 3.4 and 3.5, and is broadly distributed across the Eastern Mediterranean. *P. abyssinicum *(Sub-Group 3.1), lies between Sub-Groups 3.2 (~*P. elatius*) and 3.6 (~*P. fulvum*) (Figure 8). Vershinin *et al*. [6] have suggested that *P. abyssinicum *may have derived from a cross between *P. fulvum *and *P. elatius*. Our data support this conclusion and suggest a particular subset of *P. elatius *germplasm as the specific source for the cross. This represents a domestication event as *P. abyssinicum *is grown as a landrace crop in Ethiopa and nearby countries. The geography of this event is puzzling at first sight because Sub-Groups 3.2 and 3.6 are apparently absent from Ethiopia and Sudan (Figure 8). However, they coexist in the Western half of the Fertile Crescent (Israel/Jordan/Lebanon/Syria/Southern Turkey) and we suggest that a hybrid pea sample was collected in this area and transferred by humans to North East Africa where it was developed into the modern *P. abyssinicum*. This hypothesis is supported by the surprisingly low diversity of *P. abyssinicum *in contrast to most landrace crops [6]. We suggest that the ancestral germplasm of *P. abyssinicum *was a small sample, introducing a bottleneck into the diversity of this gene pool that still survives today.

The exact mode of derivation of *P. sativum *from wild *Pisum *is less clear but our data do provide some clues. The majority of landrace *P. sativum *is found in Groups 1 and 3, with the apparently more primitive varieties mainly present in Sub-Groups 3.2-3.5 [35,38,39]. The *P. sativum *presumptive sub-species tend to be too poorly represented in the JI Collection to allow confident Sub-Group assignment, with the possible exception of *P. transcaucasicum *with 8 accessions, 4 of which belong to Sub-Group 3.5 (Table 1). This Sub-Group shows a broad geographic distribution across Southern Eurasia (Figure 8), from Greece to Nepal which has long been recognised as an interesting source of primitive sub groups [38,39]. Sub-Groups 3.3 and 3.4 show geographic overlap with this Sub-Group (Figure 8) but are themselves much more strongly associated with particular regions (Afghanistan and Nepal/India respectively). Sub-Groups 3.4 and 3.5 both contain *P. elatius *germplasm but Sub-Group 3.3 apparently lacks it (Additional file 2). Furthermore, Sub-Groups 3.4 and 3.5 are roughly equidistant from Groups 1 and 2 which represent the bulk of modern cultivated *P. sativum *but Sub-Group 3.3 is more remote and apparently derived from Sub-Group 3.4 (Figure 6).

On the basis of the above observations we suggest tentatively the following model for *P. sativum *domestication. Wild *P. elatius *with affinity to Sub-Group 3.5 was selected by early farmers in the Fertile Crescent. This domesticate was then grown extensively, broadening its distribution across Southern Eurasia and additionally differentiated in two opposite directions. The first of these was an expansion eastwards into the Indian subcontinent and the Himalayan regions involving Sub-Group 3.4, which subsequently gave rise to the Afghan ecotypes represented by Sub-Group 3.3. We cannot exclude the possibility that wild Sub-Group 3.4 germplasm also contributed to this process and further more detailed study will be needed to test this and other aspects of our model. The second proposed diversification of primitive cultivated Sub-Group 3.5 *P. sativum *was the main domestication route that gave rise to Groups 1 and 2 which represent the mainstream of modern cultivar *Pisum*.

The only other Sub-Group with either strong support from our molecular analysis or restricted geographical localisation is Sub-Group 1.1 whose distribution is centred upon Ethiopia and Spain but whose allelic spectrum suggests that it lacks large scale introgression from any particular wild germplasm (Figure 6, Figure 8). None of the poorly supported Sub-Groups comprising *P. sativum *cultivars and landraces (Sub-Groups 1.2-1.6 and 2.1-2.1) show good resolution from other members of the same Group, in either of the diversity plots or their geographic origin. We conclude that these Sub-Groups are tenuous, with little support from this study.

A recent microsatellite based analysis of Chinese germplasm [12] has shown this material to be very diverse and with a strong geographical partitioning of this variation. It is difficult to relate our study to this work because we have only 32 Chinese accessions and virtually none are shared between the two studies. Interestingly Sub-Groups 3.1, 3.2, 3.4 and 3.5 are represented in our limited set of Chinese material but a larger proportion of our Chinese accessions are in Group 1 (but none occupy subgroup 1.1; Figure 8). Zong *et al *suggest that some of the genetic background to the Chinese material may derive from *Pisum *outside the Fertile Crescent [12]. This is consistent with our South Chinese accession JI1398 which is found in subgroup 1.3 and has the phenotypic properties of *P. elatius *(but was classified as *P. sativum *by Smith) [13]. Clearly, further work on shared germplasm sets is needed to resolve these issues.

### A new framework for Pisum core germplasm collections

The findings reported here add considerably to our understanding of the global diversity of the *Pisum *genus. The genotyping of a very broad collection of germplasm has both confirmed previous findings and defined important new distinct germplasm sets that were unsuspected. For example, Sub-Group 1.1 was not recognised previously and was not represented in the early diverse test array of 56 lines used in previous studies of the diversity of the John Innes *Pisum *Collection, hence it is not represented in Figure 5. This prompts a more detailed reassessment of not only this material but also germplasm from across other Sub-Groups with uneven representation with a view to removing concentrations of related samples such as 3.6 and introducing new unrepresented germplasm, such as Sub-Groups 1.6, 3.3 and 3.5 (Figure 5). These detailed considerations will form part of future studies and recommendations for core collections on various scales, which will include more germplasm from other collections (Manuscript in preparation).

## Conclusion

These data provide new insights into the genetic diversity and evolution of the genus *Pisum *and its spatial distribution. We have proposed and elaborated a new model for the origin of cultivated field pea involving two historically ancient but evolutionarily recent domestications of subsets of wild *Pisum *germplasm. We must point out that this model is based upon small numbers of sampled wild germplasm which may be biased but it suggests further experiments to test it. Lastly, we have produced a new framework for defining a global *Pisum *germplasm collection which will result in more rational choice of materials for future study and exploitation by agriculture.

## Authors' contributions

RJ carried out the molecular marker microarray studies and the gel based validation of marker scores. AV participated in growing the germplasm and prepared the DNAs and associated sample lists, Paul Shaw created the MySQL version of Germinate 2.0 and oversaw the modifications leading to the updated version 2.3. Jacek Grzebyta created the specific Germinate-Pea database together with its visualization, security and grouping tools. Petr Smykal performed control gel based marker experiments to test the reproducibility of the marker data. DM supervised the databasing work and development of associated informatics tools, MA had overall responsibility for selecting and growing the germplasm, coordinated the germplasm diversity aspects of the study and helped to draft the manuscript. THNE performed the marker data analysis, coordinated the activities at JIC and helped to draft the manuscript. AF coordinated the overall study, drafted the manuscript, was involved with establishing the molecular marker method and associated primer design, processed the primary microarray data to yield final marker scores, analysed the quality control experiments and selected markers to be incorporated into the analysis. All authors read and approved the final manuscript.

## Supplementary Material

Additional file 1 List of germplasm samples Click here for file

Additional file 2 **TAM microarray images for RBIP markers**. Each marker is scored in 3029 samples according to the spotting plan at http://www.personal.dundee.ac.uk/~ajflavel/Spot_Table.htm. Sample assignation to spots and correspondance between allelic states and fluorescence colours are at http://www.personal.dundee.ac.uk/~ajflavel/Spot_Table3.htm and also at the Germinate pea database [23]. Click here for file

Additional file 3 **Germinate database images**. A. Information retrieved from the Germinate-Pea database for a single accession (JI2055). This line carries associated latitude-longitude data for the sample collection site and this can be viewed by an active link to Google Maps. The Germinate-Pea database is freely accessible at [23]. B. Results of marker analysis using the 281 × 44 RBIP marker. The top image is the original TAM microarray image and a pseudo-image below records the deduced scores for all accessions. Each spot in the pseudo-image can be moused-over to show associated accession data (JI1555 is illustrated). At the bottom the graph shows the total array marker scores plotted as corrected pixel values, colour-coded by deduced score. Click here for file

Additional file 4 **Structure simulations for K values of 3, 7 and 11**. The results of 10 independent Structure runs at K = 3, K = 7 and K = 11 are shown. For each run the fractional inferred ancestry of the 3029 individuals is plotted as a histogram, with each ancestral population colour-coded. Accessions are assigned to Group N if their average representation (Q_N_) for that Sub-Group ≥ 0.5. For K = 3, accessions are ordered according to the mean representation for all runs (mean) and in Groups 1 and 2 by decreasing Q and for Group 3 by increasing Q. Admixed accessions, where Q_1_+Q_2 _= 0.5 > Q_3_, are placed between Groups 2 and 3, ordered by increasing Q_3_. At the bottom of the K = 3 Figure the fraction of missing data per accession is plotted as a black vertical bar. For K = 7 the Q plots are similarly constructed, but accession order determined by the mean of runs 8, 9 and 10 which are the most highly correlated set. For K = 11 the Q plots have accessions in the same order as for K = 3. Run 10 is replotted using three colours corresponding to ΣQ_1 _to Q_7_, Q_8_+Q_9 _and Q_10_+Q_11_ Click here for file

Additional file 5 **Reproducibility between Structure runs at different K values**. Correlations between assignments of ancestry for each K Group in 10 Structure runs with K values of 3, 7 and 11 respectively are shown. A) Correlation matrix relating all population assignments. Each rectangle bounded in grey is a single pairwise comparison between structure simulations where the value of the correlation coefficient between two Groups is represented by the colour scale as indicated. The diagonal set compares each run with itself. B) Eight pairwise comparisons are enlarged. The top four of these comprise two self comparisons and one K = 3/K = 7 shown in duplicate adjacent to these. Below these are two independent K = 3/K = 7 and K = 7/K = 7 comparisons. For the latter the diagonal represents the reproducibility between the corresponding Structure runs. C) An example of a single K = 3/K = 7 comparison. The X axis corresponds to a K = 3 run and the Y axis to a K = 7 run. The dots within the scatter graph correspond to the position of an accession in the K = 3 and K = 7 runs respectively. The position of accessions in these axes is determined by decreasing Q for each of K populations, and these are grouped into K blocks where Q is above 0.5. For each comparison there are K+1 blocks of compared accessions; the additional one comparing the admixture group where no value of Q < 0.5. A table of all pairwise correlations is available as an EXCEL file on request. Click here for file

Additional file 6 **Composition of Germplasm Structure Sub-Groups**. Numbers of accessions within Structure Sub-Groups and *Pisum *taxa are colour-coded as in Figures 4, 5, 6, 7, 8 and Figures 4, 5, 6 respectively. Click here for file

Additional file 7 **Integrity of Sub-Group 1.1 in a K = 11 Structure plot**. Structure plots of Q values are presented as described in Figure 3. Individual runs at K = 3, K = 7 and K = 11 are shown together with the Sub-Groups shown in Figure 4. The accession order is the same for all four panels. The accessions corresponding to Sub Group 1.1 are boxed in red. Click here for file
