# Supplementary figures and images for: The genetic diversity and evolution of field pea (Pisum) studied by high throughput retrotransposon based insertion polymorphism (RBIP) marker analysis
Source: BMC Evol Biol. 2010 Feb 15;10:44. doi: 10.1186/1471-2148-10-44 (PMC2834689; doi:10.1186/1471-2148-10-44)

45x8

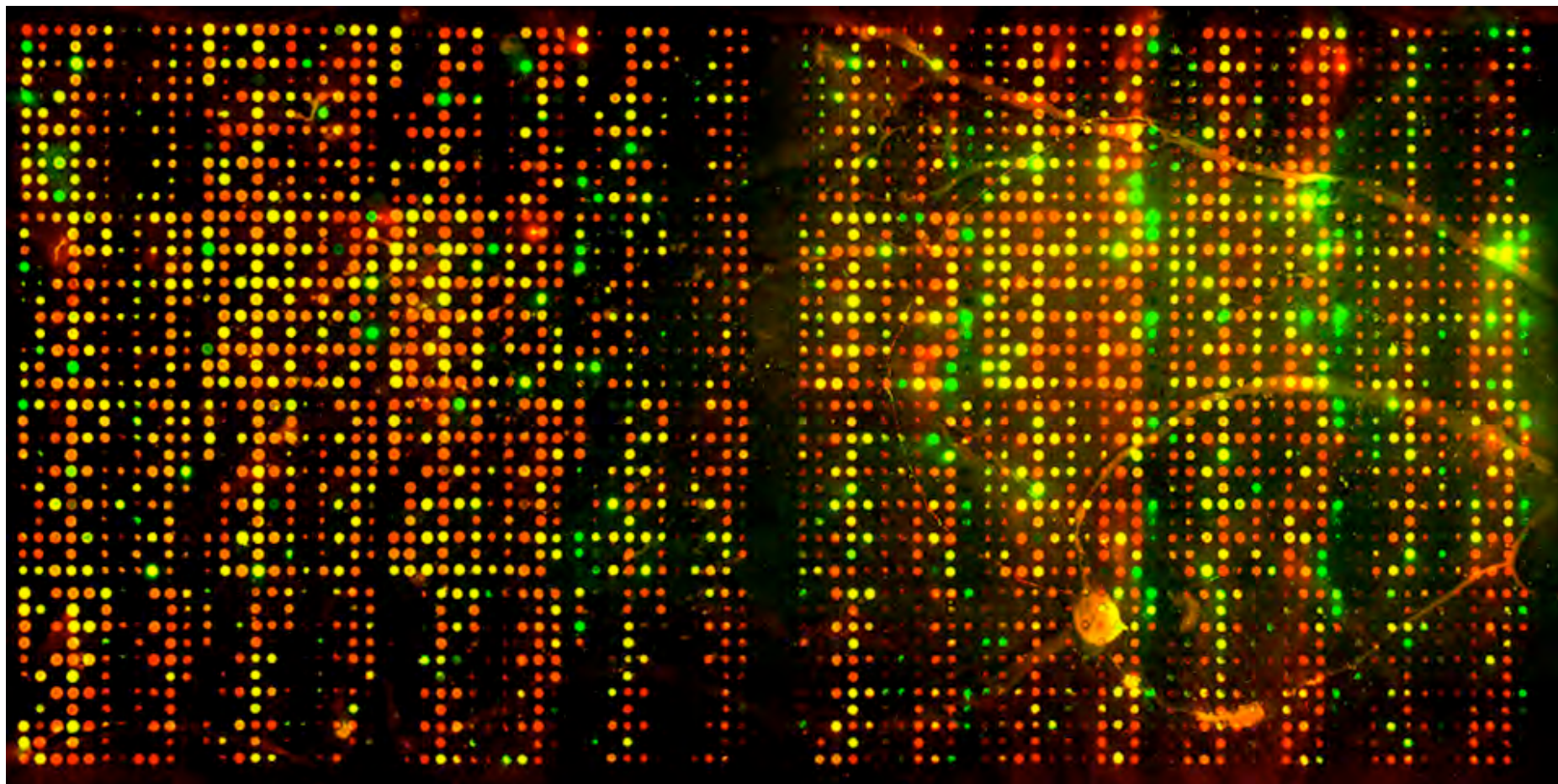

45x15

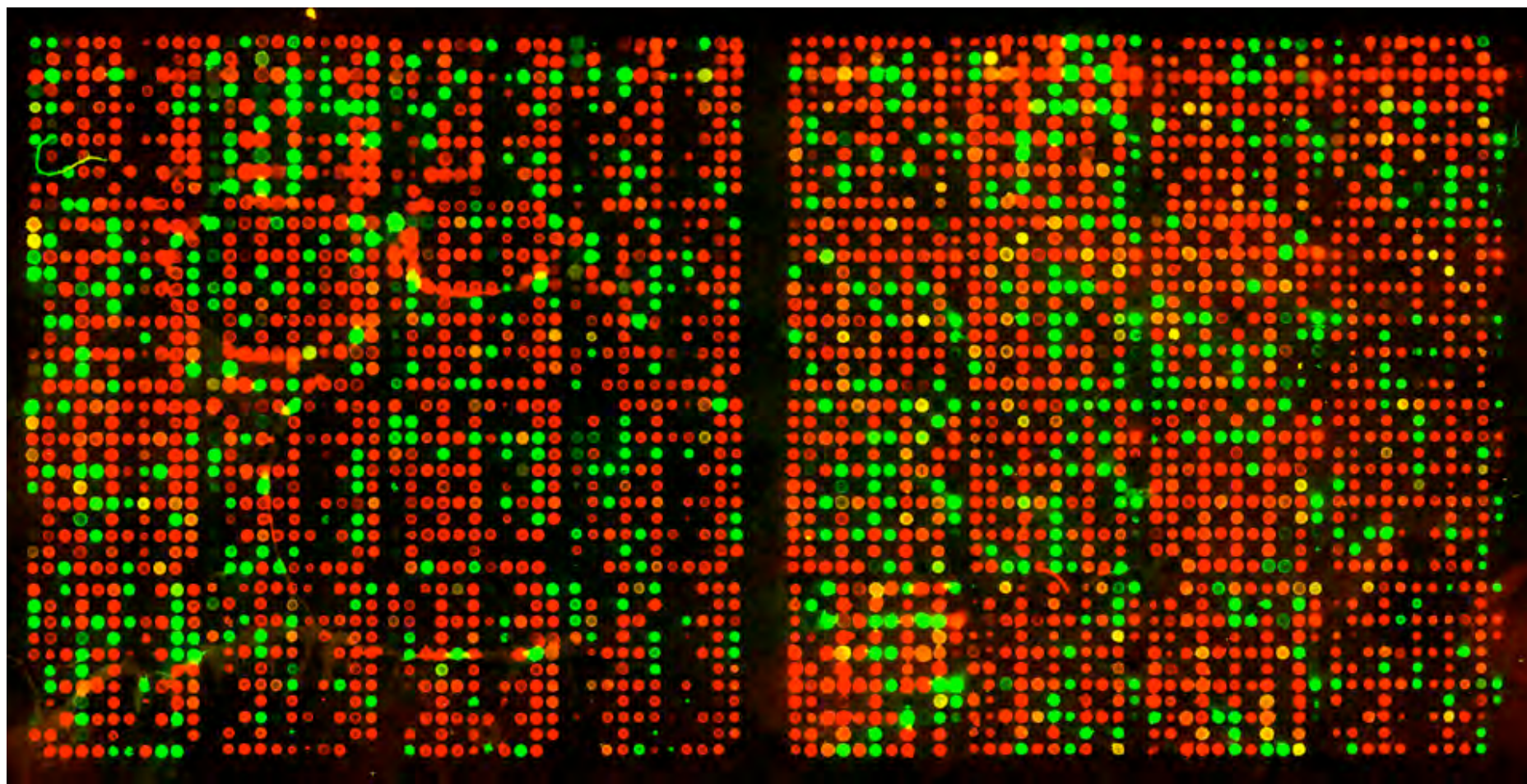

45x20

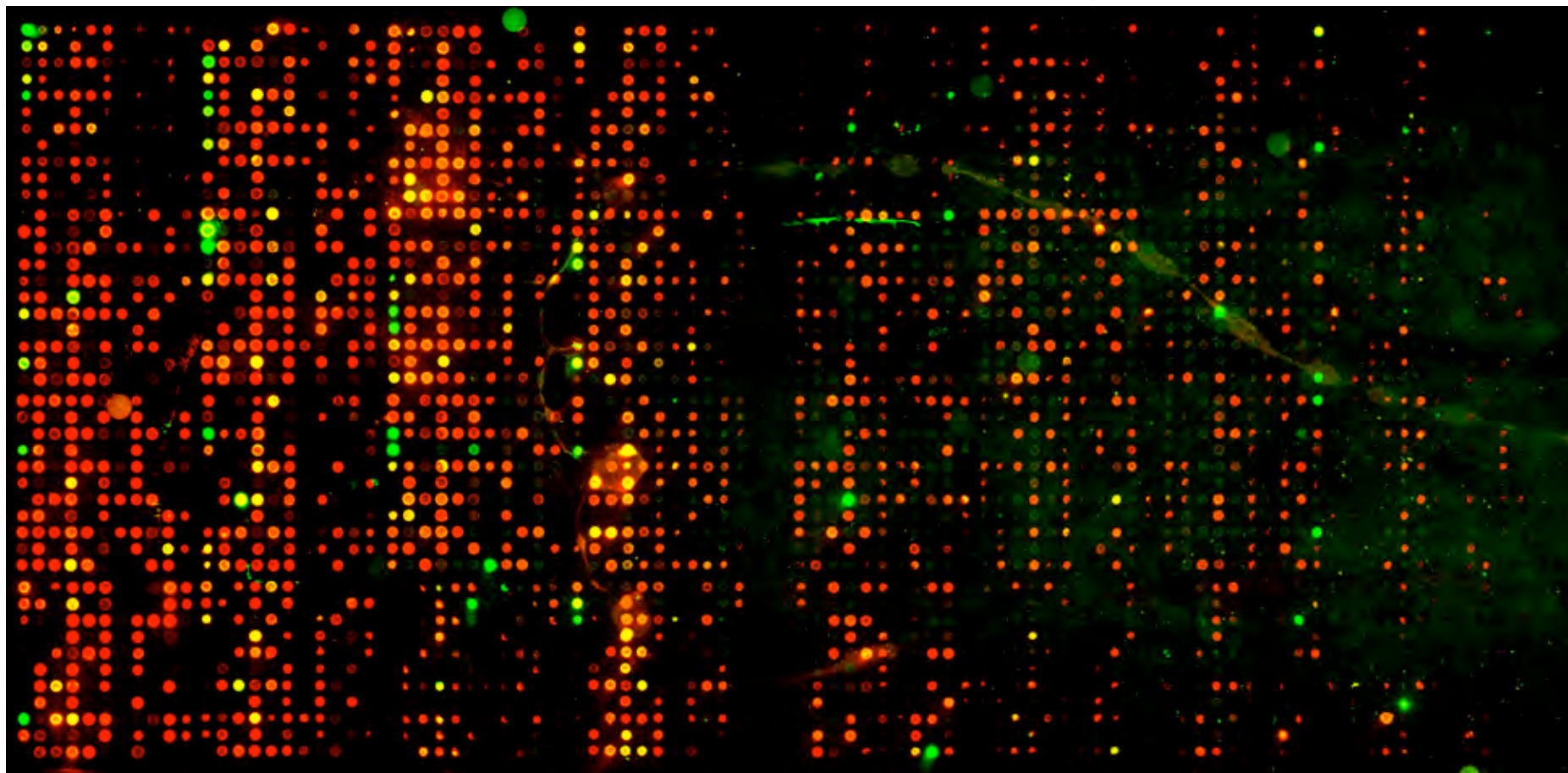



45x31

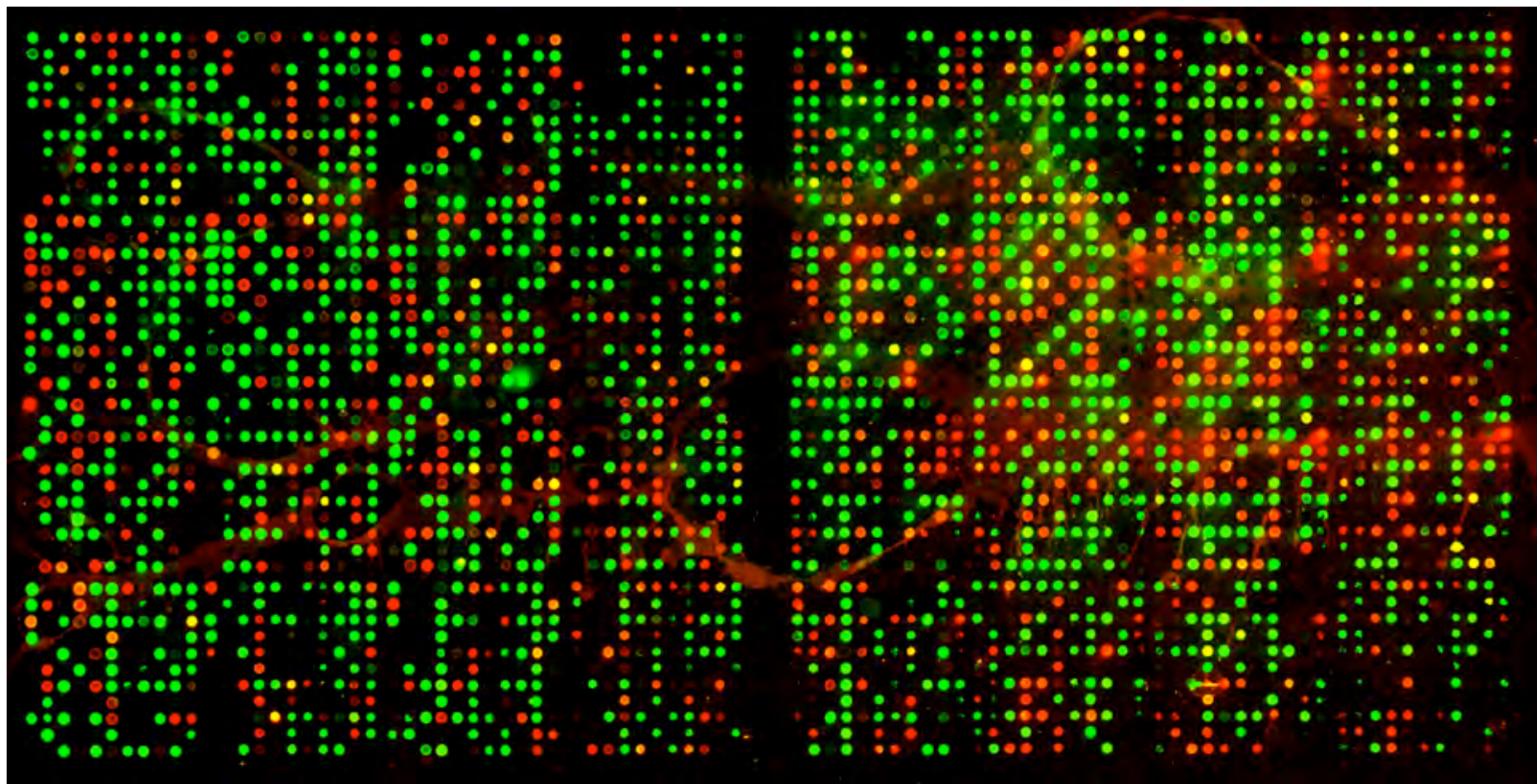

45x33

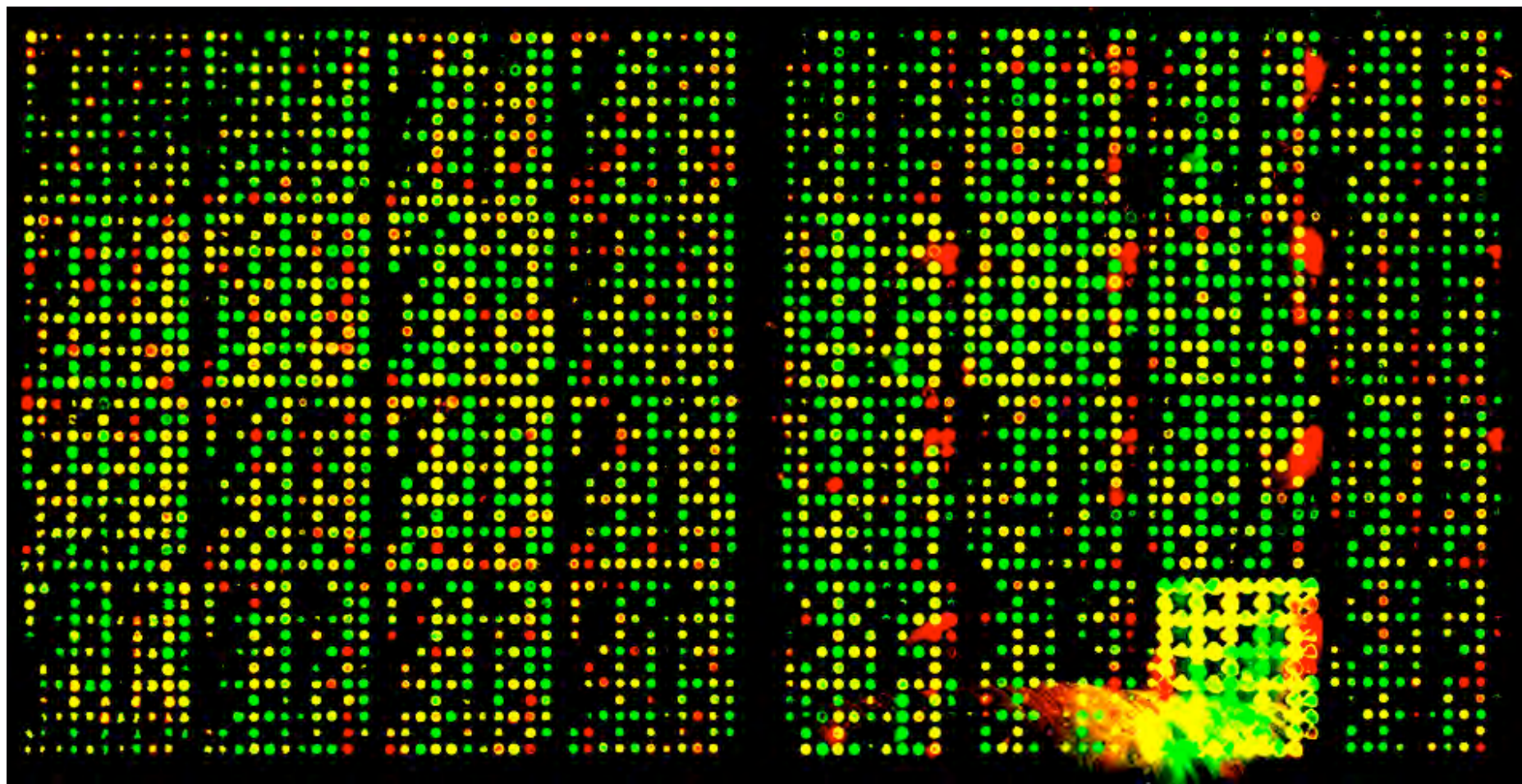

45x38

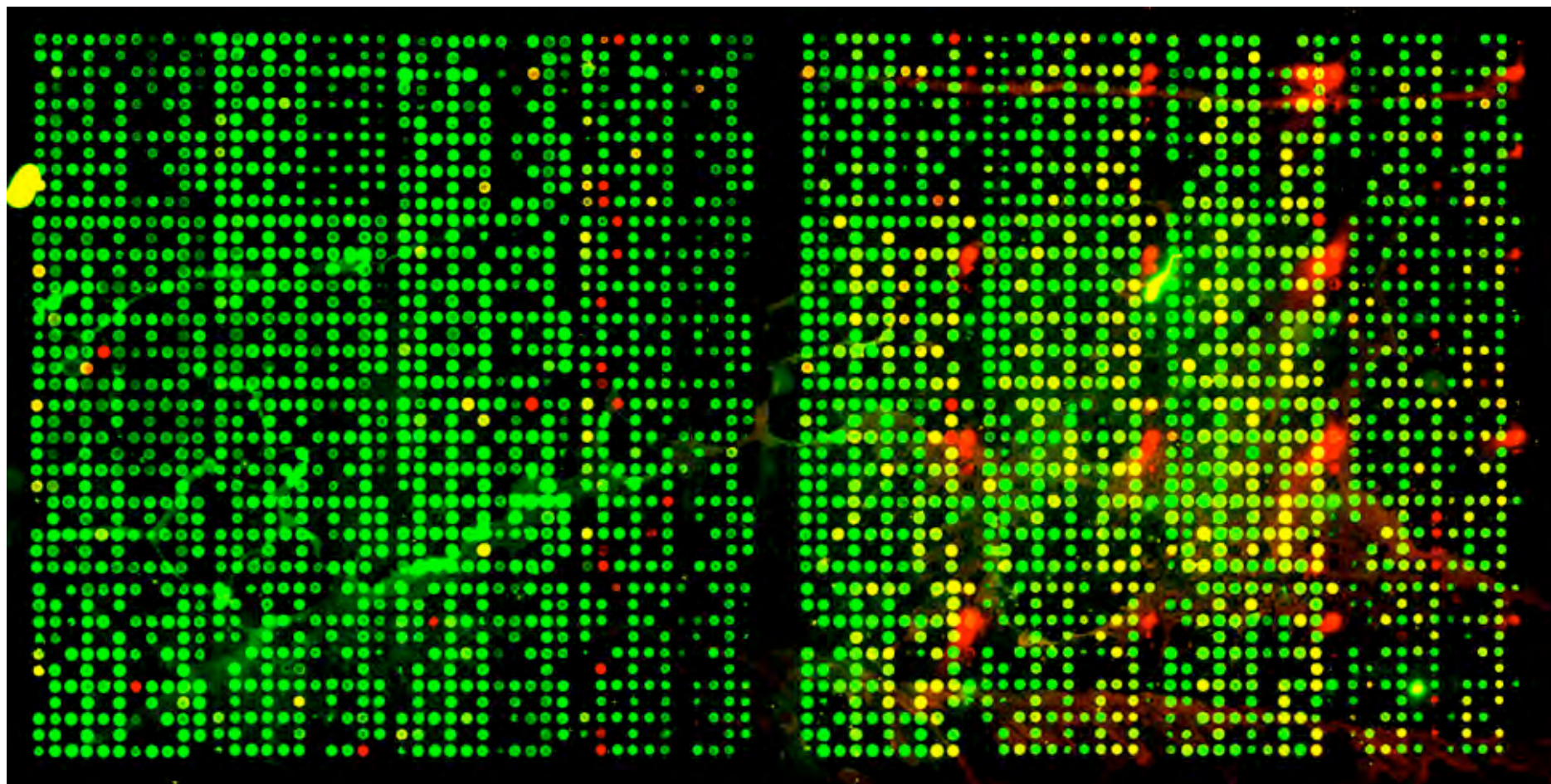





64x15

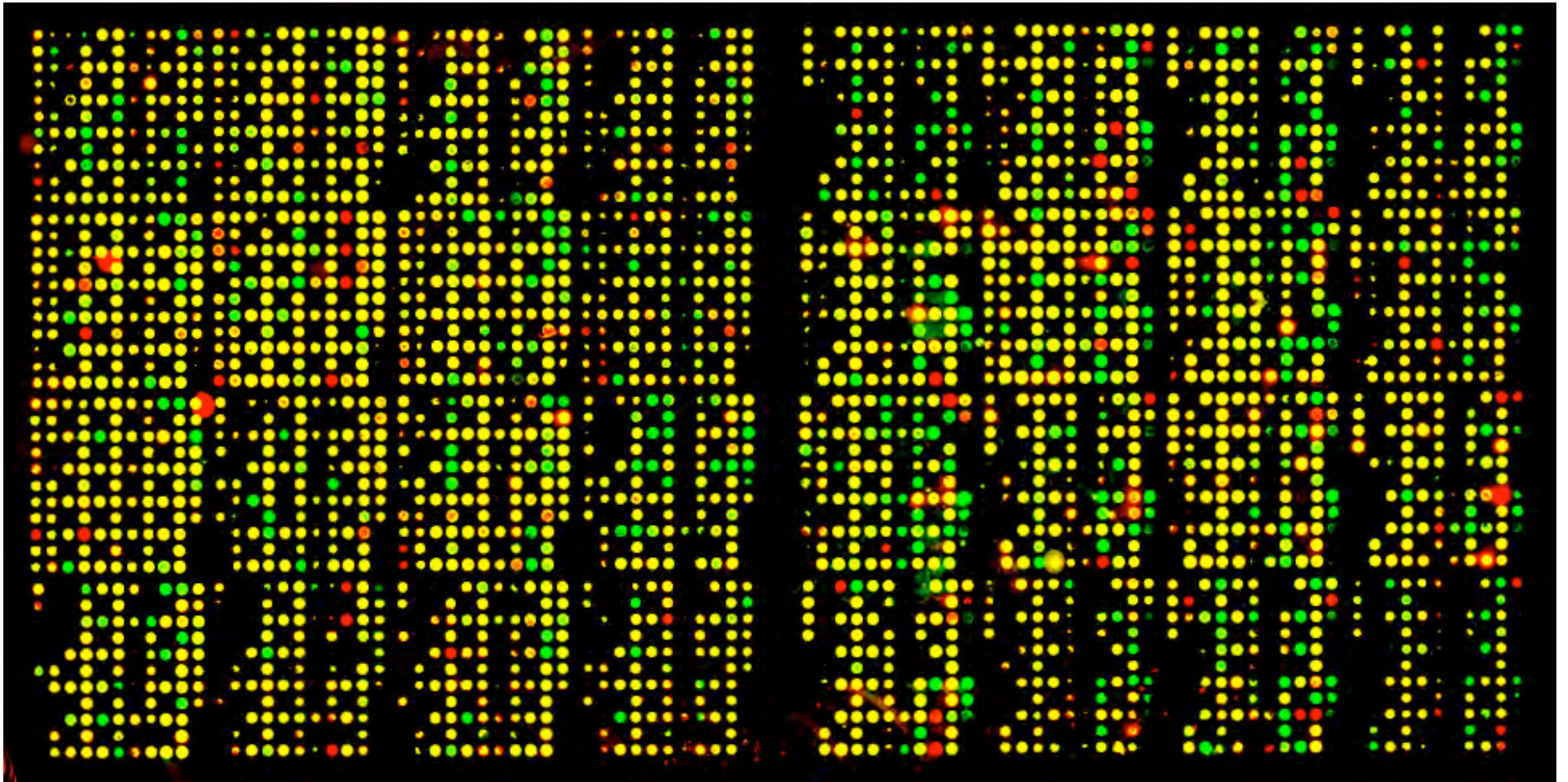



























281x16

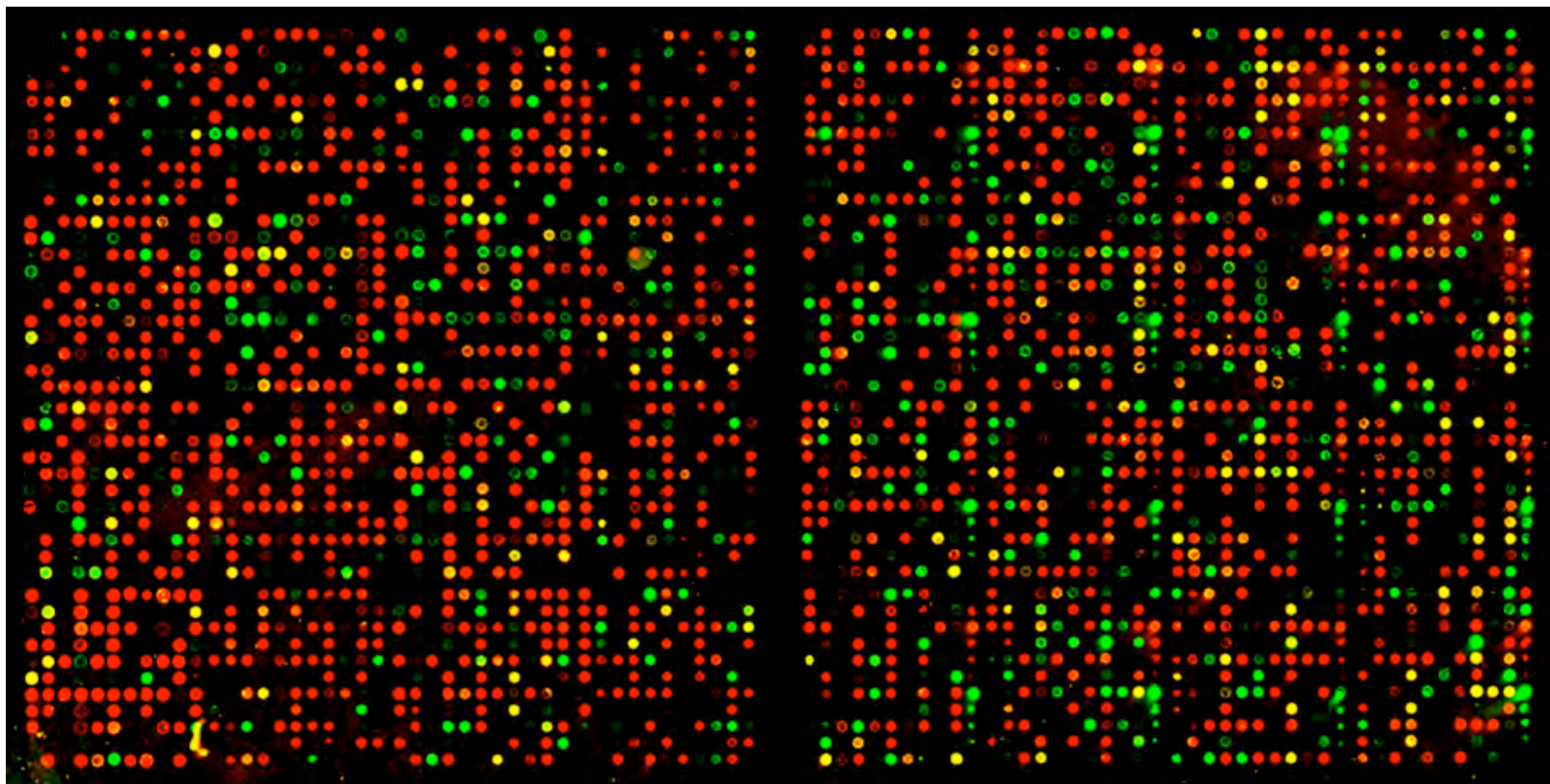

Supplement: Additional file 2 — TAM microarray images for RBIP markers. Each marker is scored in 3029 samples according to the spotting plan at http://www.personal.dundee.ac.uk/~ajflavel/Spot_Table.htm. Sample assignation to spots and correspondance between allelic states and fluorescence colours are at http://www.personal.dundee.ac.uk/~ajflavel/Spot_Table3.htm and also at the Germinate pea database [23]. [file 1471-2148-10-44-S2.PDF]

K=3

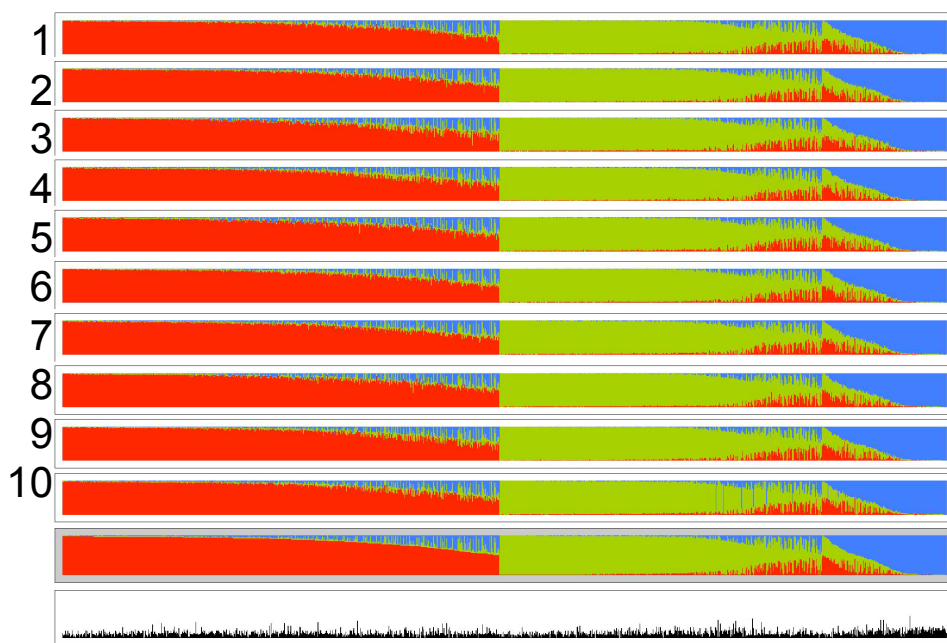

K=7

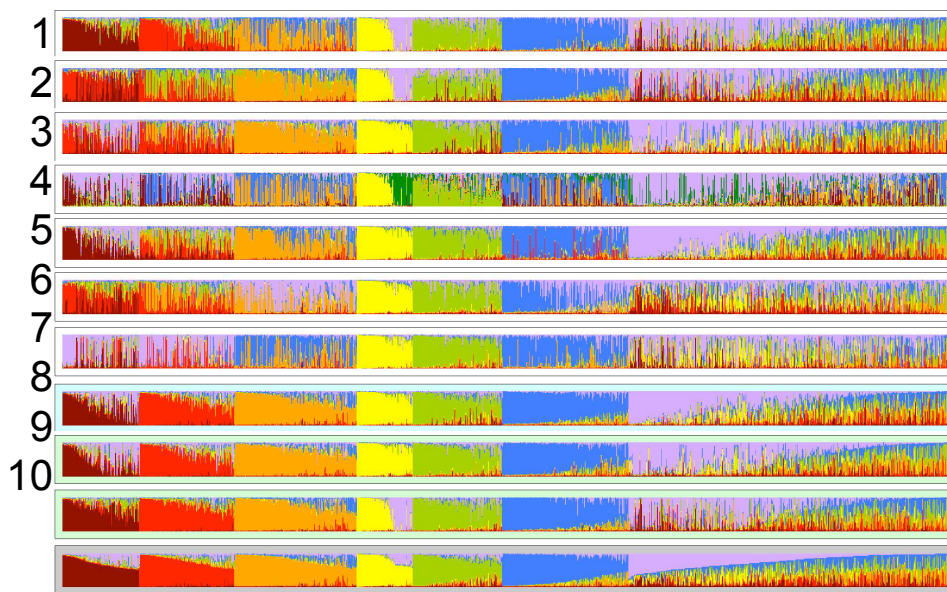

K=11

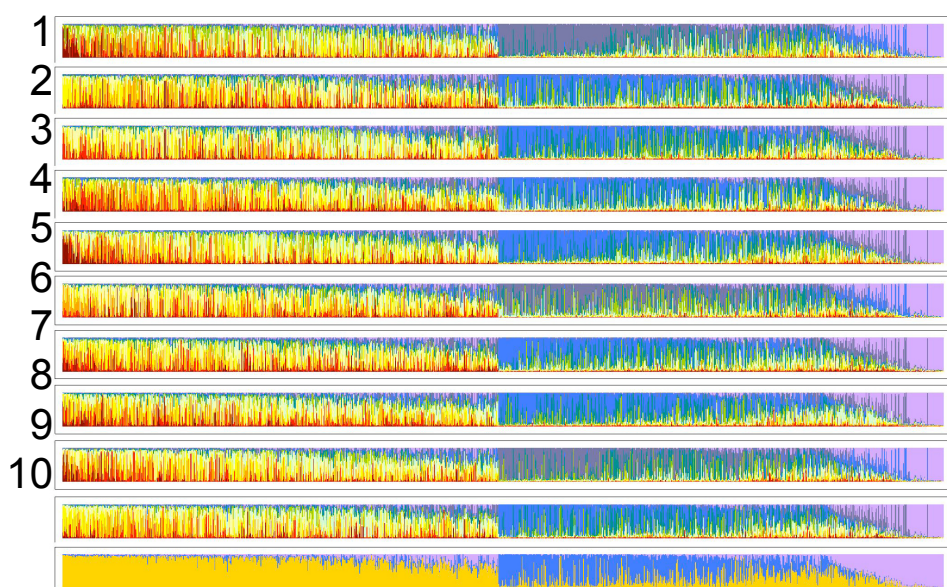

Supplement: Additional file 4 — Structure simulations for K values of 3, 7 and 11. The results of 10 independent Structure runs at K = 3, K = 7 and K = 11 are shown. For each run the fractional inferred ancestry of the 3029 individuals is plotted as a histogram, with each ancestral population colour-coded. Accessions are assigned to Group N if their average representation (QN) for that Sub-Group ≥ 0.5. For K = 3, accessions are ordered according to the mean representation for all runs (mean) and in Groups 1 and 2 by decreasing Q and for Group 3 by increasing Q. Admixed accessions, where Q1+Q2 = 0.5 > Q3, are placed between Groups 2 and 3, ordered by increasing Q3. At the bottom of the K = 3 Figure the fraction of missing data per accession is plotted as a black vertical bar. For K = 7 the Q plots are similarly constructed, but accession order determined by the mean of runs 8, 9 and 10 which are the most highly correlated set. For K = 11 the Q plots have accessions in the same order as for K = 3. Run 10 is replotted using three colours corresponding to ΣQ1 to Q7, Q8+Q9 and Q10+Q11 [file 1471-2148-10-44-S4.PDF]

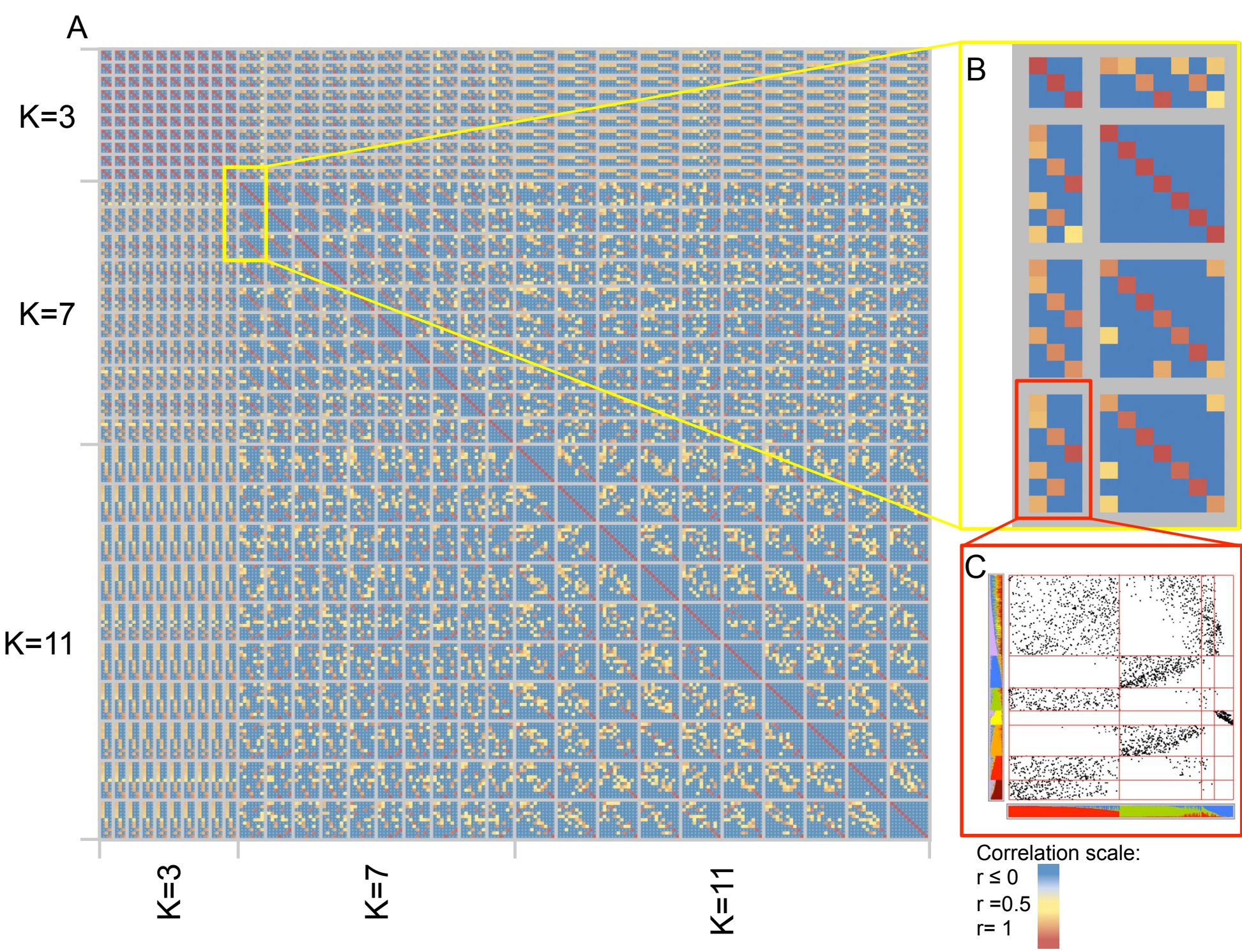

Supplement: Additional file 5 — Reproducibility between Structure runs at different K values. Correlations between assignments of ancestry for each K Group in 10 Structure runs with K values of 3, 7 and 11 respectively are shown. A) Correlation matrix relating all population assignments. Each rectangle bounded in grey is a single pairwise comparison between structure simulations where the value of the correlation coefficient between two Groups is represented by the colour scale as indicated. The diagonal set compares each run with itself. B) Eight pairwise comparisons are enlarged. The top four of these comprise two self comparisons and one K = 3/K = 7 shown in duplicate adjacent to these. Below these are two independent K = 3/K = 7 and K = 7/K = 7 comparisons. For the latter the diagonal represents the reproducibility between the corresponding Structure runs. C) An example of a single K = 3/K = 7 comparison. The X axis corresponds to a K = 3 run and the Y axis to a K = 7 run. The dots within the scatter graph correspond to the position of an accession in the K = 3 and K = 7 runs respectively. The position of accessions in these axes is determined by decreasing Q for each of K populations, and these are grouped into K blocks where Q is above 0.5. For each comparison there are K+1 blocks of compared accessions; the additional one comparing the admixture group where no value of Q < 0.5. A table of all pairwise correlations is available as an EXCEL file on request. [file 1471-2148-10-44-S5.PDF]

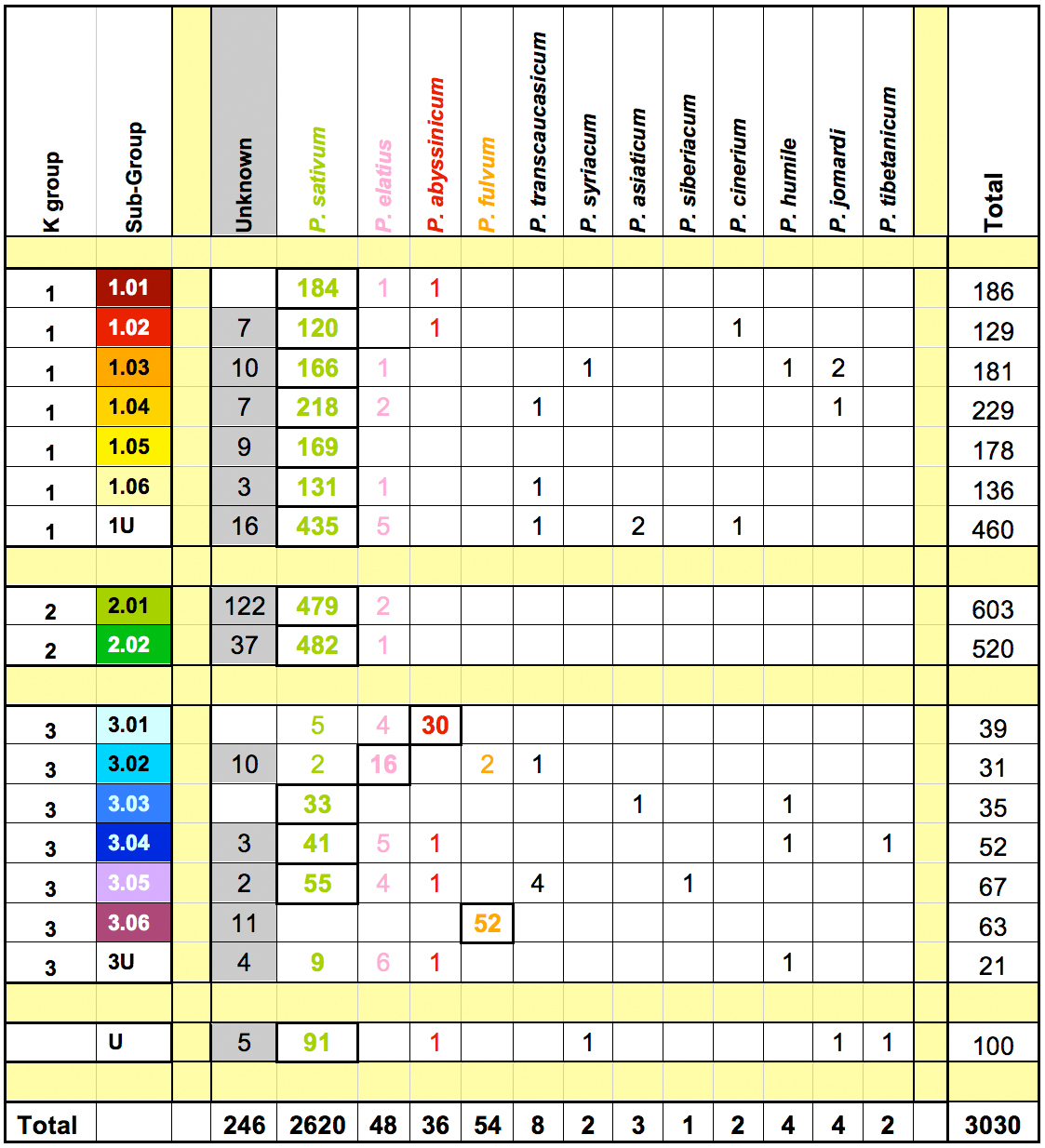

Supplement: Additional file 6 — Composition of Germplasm Structure Sub-Groups. Numbers of accessions within Structure Sub-Groups and Pisum taxa are colour-coded as in Figures 4, 5, 6, 7, 8 and Figures 4, 5, 6 respectively. [file 1471-2148-10-44-S6.JPEG]

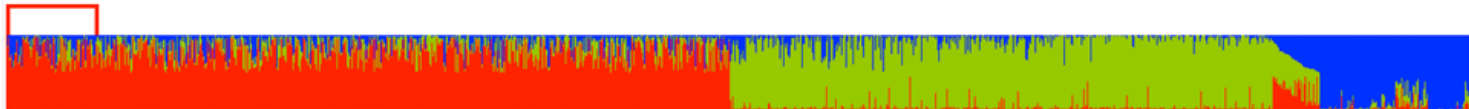

K=3

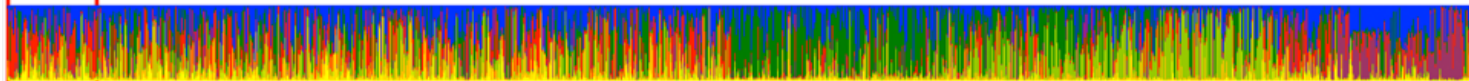

K=7

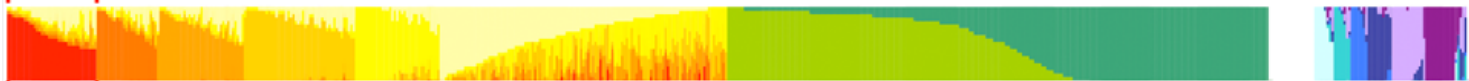

K=3 Sub-Groups

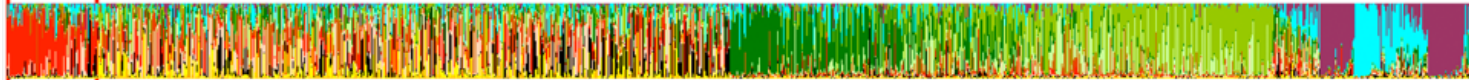

K=11

Supplement: Additional file 7 — Integrity of Sub-Group 1.1 in a K = 11 Structure plot. Structure plots of Q values are presented as described in Figure 3. Individual runs at K = 3, K = 7 and K = 11 are shown together with the Sub-Groups shown in Figure 4. The accession order is the same for all four panels. The accessions corresponding to Sub Group 1.1 are boxed in red. [file 1471-2148-10-44-S7.PDF]
