# Supplementary material for: The genetic diversity and evolution of field pea (Pisum) studied by high throughput retrotransposon based insertion polymorphism (RBIP) marker analysis
Source: BMC Evol Biol. 2010 Feb 15;10:44. doi: 10.1186/1471-2148-10-44 (PMC2834689; doi:10.1186/1471-2148-10-44)
Supplement: Additional file 3 — Germinate database images. A. Information retrieved from the Germinate-Pea database for a single accession (JI2055). This line carries associated latitude-longitude data for the sample collection site and this can be viewed by an active link to Google Maps. The Germinate-Pea database is freely accessible at [23]. B. Results of marker analysis using the 281 × 44 RBIP marker. The top image is the original TAM microarray image and a pseudo-image below records the deduced scores for all accessions. Each spot in the pseudo-image can be moused-over to show associated accession data (JI1555 is illustrated). At the bottom the graph shows the total array marker scores plotted as corrected pixel values, colour-coded by deduced score. [file 1471-2148-10-44-S3.PDF]

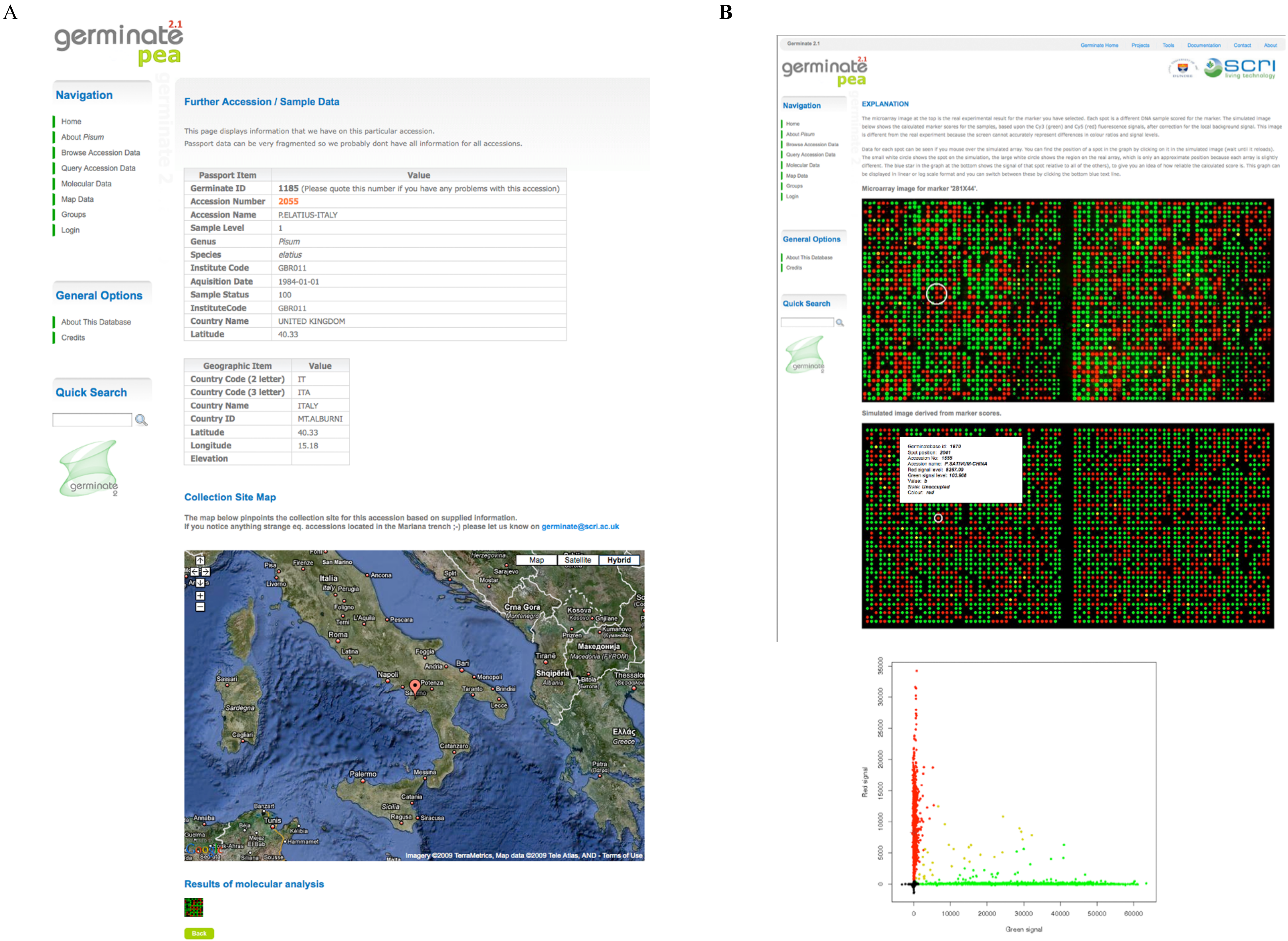
A
germinate 2.1
pea
Navigation
Home
About Pisum
Browse Accession Data
Query Accession Data
Molecular Data
Map Data
Groups
Login
General Options
About This Database
Credits
Quick Search
germinate 2
Further Accession / Sample Data
This page displays information that we have on this particular accession.
Passport data can be very fragmented so we probably dont have all information for all accessions.
Passport Item | Value
Germinate ID | 1185 (Please quote this number if you have any problems with this accession)
Accession Number | 2055
Accession Name | P.ELATIUS-ITALY
Sample Level | 1
Genus | Pisum
Species | elatius
Institute Code | GBR011
Aquisition Date | 1984-01-01
Sample Status | 100
InstituteCode | GBR011
Country Name | UNITED KINGDOM
Latitude | 40.33
Geographic Item | Value
Country Code (2 letter) | IT
Country Code (3 letter) | ITA
Country Name | ITALY
Country ID | MT.ALBURNI
Latitude | 40.33
Longitude | 15.18
Elevation |
Collection Site Map
The map below pinpoints the collection site for this accession based on supplied information.
If you notice anything strange eq. accessions located in the Mariana trench ;-) please let us know on germinate@scri.ac.uk
Map
Satellite
Hybrid
Imagery ©2009 TerraMetrics, Map data ©2009 Tele Atlas, AND - Terms of Use
Results of molecular analysis
Back
B
Germinate 2.1
Germinate Home
Projects
Tools
Documentation
Contact
About
germinate 2.1
pea
scri
living technology
Navigation
General Options
Quick Search
EXPLANATION
Microarray image for marker '281X44'.
Simulated image derived from marker scores.
Red signal
Green signal
0
5000
10000
15000
20000
25000
30000
35000
40000
50000
60000
